# Separation and Valorization of Chlorine From Tail Gas Streams

**DOI:** 10.1002/anie.3641857

**Published:** 2026-08-11

**Authors:** Gesa H. Dreyhsig, Merlin Kleoff, Patrick Voßnacker, Elisabeth Dorn, Jacob H. Richter, Sebastian Riedel

**Affiliations:** ^1^ Institute of Chemistry and Biochemistry Inorganic Chemistry Freie Universität Berlin Berlin Germany

**Keywords:** adsorption, chlorine, industrial chemistry, polymers, sustainable chemistry

## Abstract

The chemical industry generates chlorine‐containing tail gas streams on a million‐ton scale. Following the established linear approach, these streams are neutralized, consuming large quantities of caustic soda (NaOH), thereby producing waste streams of lower value. To overcome this unsustainable linear approach, we developed a circular process to recycle chlorine from tail gas streams. The process is enabled by an ion exchange resin [IEx]Cl that selectively adsorbs chlorine in the presence of other gases, including oxygen, nitrogen, carbon dioxide, and hydrogen, while forming the corresponding trichloride [IEx][Cl_3_] by halogen bonding. Upon application of heat (80°C) and reduced pressure the adsorbed chlorine is efficiently released, and the ion exchange resin [IEx]Cl is regenerated. Importantly, the adsorbed chlorine can also be directly converted with ethylene or carbon monoxide into the base chemicals 1,2‐dichloroethane or phosgene, respectively. In this way, chlorine from tail gas streams can be recycled and valorized in an integrated process supporting the transformation of the chemical industry toward a more circular economy.

## Graphical abstract

A commercially available ion exchange resin was found to adsorb chlorine gas selectively in the presence of other gases reversibly forming the corresponding trichloride species [IEx][Cl_3_]. This discovery was telescoped to an efficient process for the recycling and valorization of chlorine from industrially abundant tail gas streams to base chemicals.

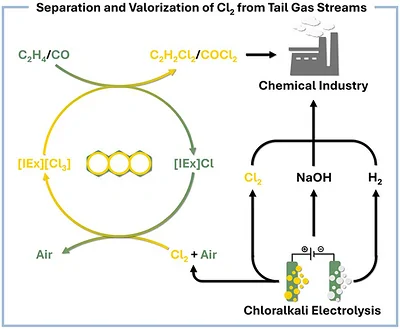

Climate change, along with growing political and societal pressure, has prompted the chemical industry to reconsider and redesign its traditional processes [1, 2, 3]. This requires not only the substitution of fossil resources, but also the recycling of industrial side streams, transforming linear processes into a circular economy [1, 4, 5, 6]. Particularly relevant are energy‐intensive processes, such as the chloralkali electrolysis, which produces chlorine (Cl_2_), caustic soda (NaOH), and hydrogen (H_2_) and accounts for approximately 0.8% of total electricity consumption in Europe (Supporting Information) [2, 7, 8, 9, 10]. Meanwhile, Cl_2_ is an indispensable base chemical required for the production of more than 50% of all industrial chemicals and polymers [11, 12, 13]. Because of its high toxicity and corrosive properties, chlorine handling is subject to strict safety measures and regulatory requirements, including the use of adsorber or scrubber systems to capture accidentally released Cl_2_ gas [12, 14, 15]. In addition to this safety function, these systems are used to clean chlorine‐containing tail gas streams primarily formed during Cl_2_ production via chloralkali electrolysis (CAE) [7, 16, 17]. Depending on previous purification steps such as low‐temperature condensation, these streams contain up to 40 mol% of Cl_2_ in air, together with traces of hydrogen (H_2_) and carbon dioxide (CO_2_) [16, 18, 19, 20]. Further recovery of this chlorine fraction would require very low temperatures, making it extremely energy‐intensive and economically unattractive [18, 21, 22]. Consequently, the chlorine content in tail gas streams is essentially inaccessible for downstream processes, leading to an accumulated Cl_2_ loss of 2–3% (corresponding to 0.016–0.024% of European electricity, which is the electricity provided by a small gas turbine power plant) [16, 17, 23, 24, 25].

To address these economically and ecologically impactful losses, Hulme invented a recovery process in 1956 using tetrachloromethane (CCl_4_) as an absorbing solvent [7, 16, 21, 26]. This approach was, however, later banned due to the high ozone depletion potential of CCl_4_ under the 1990 London Amendments of the Montreal Protocol (Figure 1B) [27]. Hooker and coworkers reported an aqueous counter‐current scrubber process capable of absorbing and partially recycling chlorine from gas mixtures [28]. Its practical use, however, is limited by undesired co‐absorption of carbon dioxide and by corrosion of the scrubber system [16, 26, 29]. Typically, this process is nowadays complemented with alkaline scrubbing utilizing caustic soda to efficiently neutralize chlorine but also carbon dioxide, allowing no complete separation (Figure 1A) [9, 16, 30]. In addition, this generates huge quantities of sodium hypochlorite (NaOCl) and sodium chloride (NaCl) as by‐products, which are of lower value [21]. An alternative concept was introduced by Hägg and coworkers using membranes to separate chlorine from other gases, allowing its recycling, yet no material was found showing sufficient durability, permeability, and selectivity (Figure 1C) [23, 31, 32, 33, 34, 35, 36].

**FIGURE 1:**
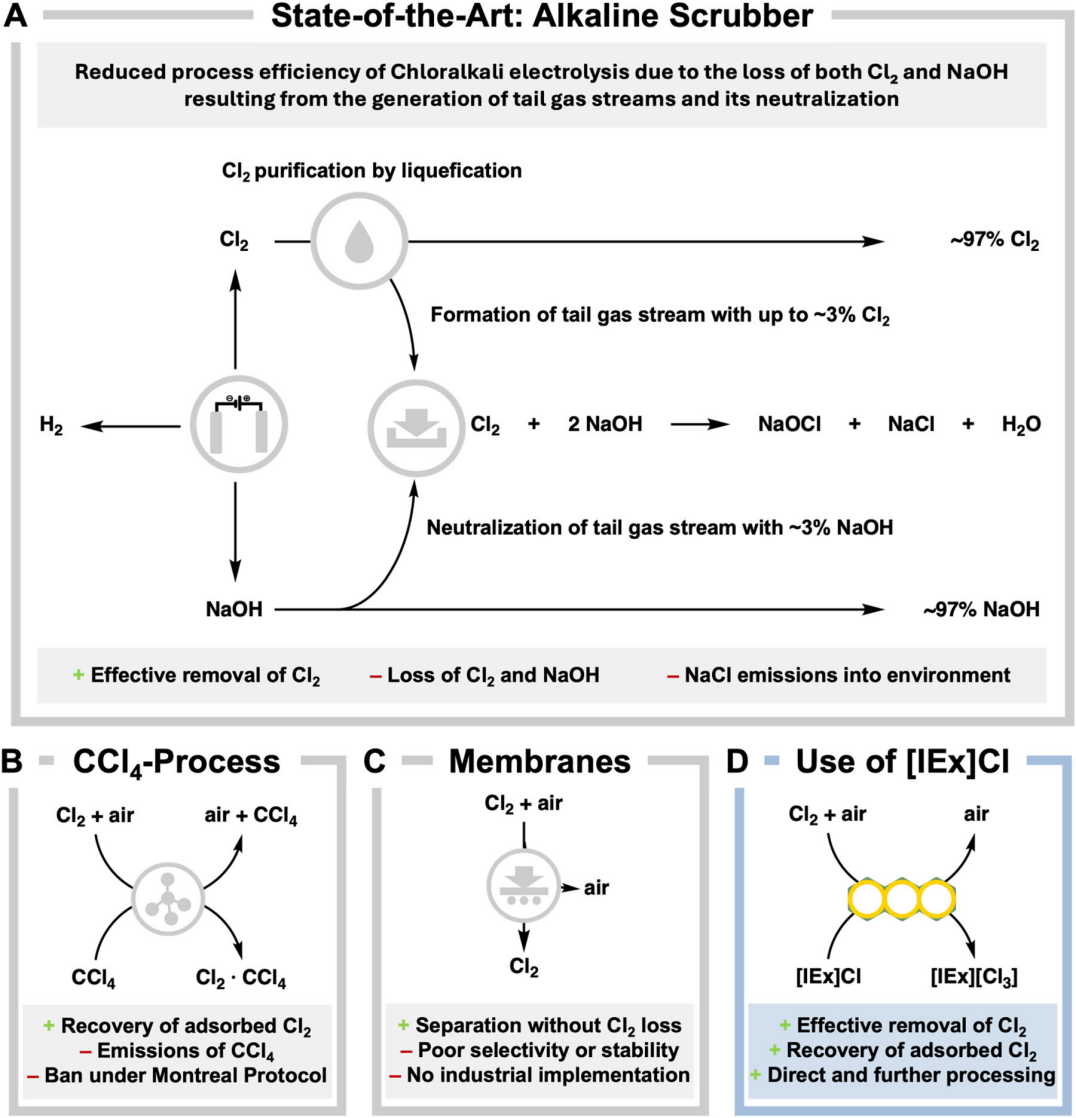
Different methods to treat chlorine‐containing tail gas streams. (A) State of the art: Loss of caustic soda (NaOH) and chlorine (Cl_2_) in alkaline scrubbers through formation of sodium hypochlorite (NaOCl), sodium chloride (NaCl), and water (H_2_O). (B) Cl_2_‐Recovery by its dissolution in prohibited tetrachloromethane (CCl_4_). (C) Use of membranes to separate Cl_2_ from air. (D) Polymer‐based ion exchange resins ([IEx]Cl) for the effective and selective removal and recovery of Cl_2_ from tail gas streams introduced by this work.

More recently, Riedel and coworkers have shown that Cl_2_ can efficiently and safely be stored in form of the ionic liquid (IL) triethylmethylammonium trichloride ([NEt_3_Me][Cl_3_]) which can also serve as chlorination agent to prepare, for example, phosgene by its reaction with carbon monoxide (CO) [2, 12, 14, 37]. Translating this concept to polymers, Haag, Riedel, and coworkers used polyethyleneimine‐derived cryogels to selectively adsorb Cl_2_, but this approach relies on a non‐commercially available material with limited stability and slow adsorption and release kinetics [38]. Inspired by early work of Manescalchi and coworkers, we envisioned to use a polystyrene‐based ion exchange resin for the reversible adsorption and separation of Cl_2_ followed by its further upcycling to the base chemicals 1,2‐dichloroethane and phosgene, enabling the recycling and valorization of Cl_2_ from tail gas streams (Figure 1D) [39, 40].

At the outset, we selected a commercially available polystyrene‐based ion exchange resin in the chloride form ([IEx]Cl) to investigate its ability to adsorb chlorine gas in comparable manner to known polyhalide systems. In initial batch experiments, we exposed the resin to gaseous Cl_2_ and noticed a rapid and exothermic adsorption accompanied by a color change from off‐white to pale yellow. Notably, no adsorption of nitrogen, oxygen, hydrogen, or carbon dioxide could be observed, allowing a selective adsorption of chlorine in the presence of these compounds commonly present in CAE tail gas streams. To investigate potential limitations of this system in more detail, we performed additional adsorption experiments with further compounds used or present in combination with Cl_2_. While observing an adsorption for hydrogen chloride (HCl) [41] and 2‐chloroaniline [42], this was not the case for 1‐chlorobutane [43] or chlorobenzene [44]. This indicates a correlation between the adsorption behavior and the hydrogen bond donor ability of the compound (Supporting Information).

The Cl_2_ storage capacity of the resin was determined gravimetrically by loading with Cl_2_ until atmospheric pressure was reached. Although lower than for the IL [NEt_3_Me][Cl_3_] (0.79 g Cl_2_/g of [NEt_3_Me]Cl), the presented system revealed a relatively high storage capacity of 0.36 g Cl_2_/g of [IEx]Cl when compared to other solid systems as, for example, polyethyleneimine‐derived cryogels (0.22–0.26 g Cl_2_/g of cryogel) and benefits from its commercial availability [2, 14, 38]. Raman spectroscopic measurements of the loaded material revealed the presence of trichloride species [Cl_3_]^–^ indicating the formation of the ion exchange resin trichloride [IEx][Cl_3_], probably enabled by halogen bonding of Cl^–^ to Cl_2_ as is also the case, for example, in [NEt_3_Me][Cl_3_] (Figure 2B and Supporting Information). As these bonds are comparably strong with 99 ± 5 kJ/mol for [Cl_3_]^−^ (gas phase), they might be the reason for the relatively high selectivity to adsorb chlorine and its separation from other compounds [14]. In analogy to the previously studied ionic liquids, this compound exists in an equilibrium with the chloride form [IEx]Cl and chlorine gas (Cl_2_), which can be shifted by heat or under reduced pressure to release the previously adsorbed chlorine (Figure 2B) [14]. To investigate this in more detail, we measured the vapor pressure curve of the loaded material, showing a pressure increase with higher temperatures, reaching a vapor pressure of 6.0 bar at 100°C (Figure S7). On the other hand, even at a low chlorine vapor pressure of 100 mbar, a reasonably high storage capacity of 0.22 g Cl_2_/g of [IEx]Cl was noted, emphasizing the possibility to selectively adsorb chlorine at low concentrations from gas mixtures (Figure S6).

**FIGURE 2:**
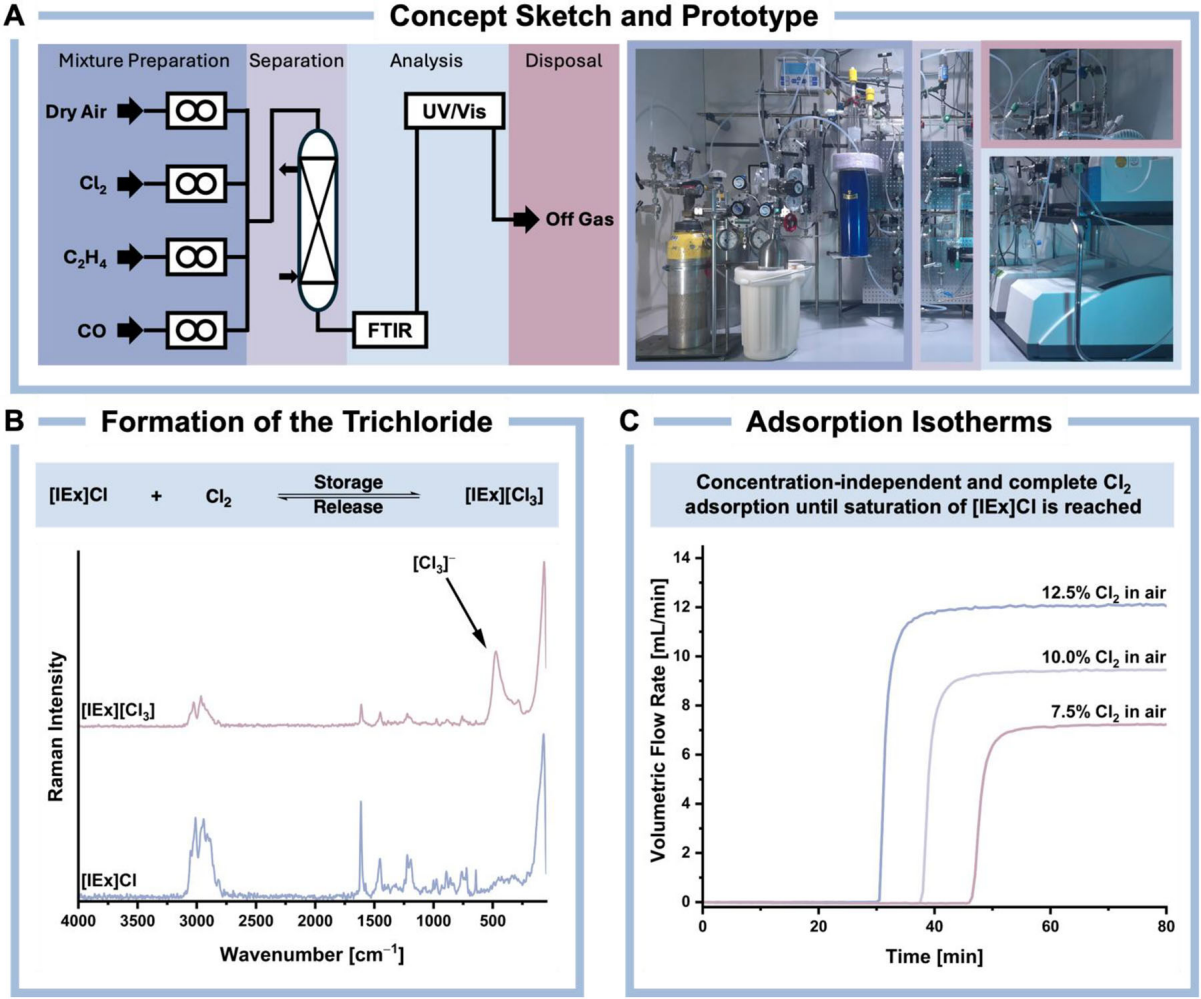
A) Design and construction of a self‐assembled setup for adsorption and desorption experiments. (B) Raman spectroscopic investigation of the chlorine‐loaded ion exchange resin ([IEx][Cl_3_]), revealing the presence of a trichloride species at $\tilde{v}$ = 475 cm^−1^. (C) Adsorption isotherms of various concentrations of chlorine/air mixtures analyzed by UV–vis spectroscopy at the outlet of the adsorption column.

Next, we analyzed the long‐term stability of the used material in cycle tests. For this, we repeatedly loaded and unloaded the resin with gaseous Cl_2_ and determined its storage capacity over 10 cycles, showing no significant change in performance (Figure S1). Furthermore, no swelling of the resin was observed even over prolonged exposure to chlorine gas, which is in accordance with observations of related systems [45, 46, 47].

These preliminary results set the base for investigating the separation of Cl_2_ from other gases in an industrially more applicable flow approach using a tailor‐made flow platform, consisting of commercially available and 3D‐printed parts (Figure 2A). The center of this platform is a temperature‐controlled glass column filled with the ion exchange resin [IEx]Cl, having a diameter of 1 cm and a height of 15 cm. It served as a packed bed reactor, which can be exposed to reduced pressure and various gas mixtures premixed by mass flow controllers. At the outlet of the column, the passed gas mixtures are analyzed spectroscopically by UV–Vis or IR spectroscopy (Figures S2 and S3).

In a first row of experiments, various mixtures of Cl_2_ and air (chlorine content 7.5%–12.5%) were passed through the packed bed reactor at a combined flow rate of 100 mL/min. Analysis of the outlet stream revealed a quantitative chlorine adsorption at this flow rate for the first 46 min using a chlorine concentration of 7.5%, for the first 38 min using a chlorine concentration of 10%, and for the first 30 min using a chlorine concentration of 12.5%. After these respective time periods, the ion exchange resin reached saturation (Figure 2C). A lower flow rate (50 mL/min, 10% chlorine concentration) increased the time until the adsorber reached saturation to 75 min, while raising the flow rate to 150 mL/min resulted in a quicker saturation after 23 min; yet, both flow rates did not influence significantly the adsorption efficiency of the adsorber (Figure S16). Additional adsorption experiments at a flow rate of 100 mL/min and different temperatures revealed a faster chlorine saturation of the adsorber after 26 min at 80°C compared to 50 min at 0°C. Consequently, the storage capacity halves when the temperature is raised from 0°C to 80°C (Figure S17). These results prove the capability of the ion exchange resin to serve as an efficient and selective adsorber for chlorine‐containing tail gas streams.

Next, we aimed for efficient options to release the adsorbed chlorine while regenerating the ion exchange resin. As with rising temperature, an increased vapor pressure was measured (Figure S7), we investigated the thermal chlorine release from the loaded [IEx][Cl_3_]. Indeed, when the previously loaded (0.24 g Cl_2_/g of [IEx]Cl) ion exchange resin was exposed to reduced pressure and a temperature of 80°C (abundant process heat at industrial sites) for 1 h, 71% of the adsorbed chlorine could be released, while a complete release was observed under reduced pressure at 20°C and over 16 h.

To further pursue the idea of sustainability, we envisioned to spare the energy‐consuming step of chlorine release by directly converting the adsorbed chlorine to valuable base chemicals, thereby shifting the paradigm of thermal desorption to chemical valorization. Inspired by previous work, we anticipated reacting the adsorbed chlorine with ethylene to 1,2‐dichloroethane (ethylene dichloride, EDC), a common precursor of polyvinyl chloride (PVC), which is produced on an annual scale of 34.5 million tons globally [2, 12, 37, 48].

To test the hypothesis, we loaded the ion exchange resin using a simulated tail gas stream with a chlorine concentration of 10% in air over 15 min with a flow of 100 mL/min at 20°C to achieve a gradual loading of the adsorber column and to prevent partial loss of chlorine during the reaction step. Then, the gas feed was switched to 3.5% ethylene in air to temper the exothermicity of the reaction. At the outlet of the adsorber column, EDC but no chlorine was detected by IR and UV–vis spectroscopy, respectively, indicating complete conversion. Interestingly, no reaction occurred when just ethylene and chlorine were combined without the presence of the polymer‐based adsorber material, indicating an increase of reactivity for [IEx][Cl_3_]. The cycle of chlorine adsorption and conversion to EDC was repeated three times to simulate a continuous industrial operation. By integration of the IR‐derived time‐dependent volumetric flow rates, the yield of EDC with respect to chlorine was found to reach 56% after the first cycle, 96% after the second cycle, and 98% after the third cycle (Figure S20). The lower yield of the first cycle can be attributed to a partial adsorption of EDC by the ion exchange resin, as supported by batch experiments (Supporting Information); after the first cycle, the adsorber is saturated with EDC, and any additional EDC formed is completely released, as reflected by almost quantitative yields.

Next, we shifted our attention to the conversion of the adsorbed chlorine to the base chemical phosgene (COCl_2_), which is produced on an annual scale of 12 million tons worldwide and is mainly used for the manufacturing of polyurethanes, polycarbonates, and fine chemicals [49]. In our previous work, we could show the synthesis of phosgene by the insertion of carbon monoxide into an elongated Cl─Cl bond of the trichloride‐based ionic liquid [NEt_3_Me][Cl_3_] [37]. We anticipated that this process could be translated to the adsorber system [IEx][Cl_3_], converting the adsorbed chlorine to phosgene while regenerating [IEx]Cl. However, under the same conditions as applied for the reaction of ethylene to EDC, phosgene was formed only in low yields of 8%–26% (Figure S21). By adjusting the chlorine loading of the adsorber and increasing the reaction temperature to 80°C, a quantitative yield of phosgene in average over three adsorption–conversion cycles was achieved according to integration of the IR‐derived time‐dependent volumetric flow rates. In the reaction course, an initial fast reaction of carbon monoxide with chlorine to phosgene was detected by IR spectroscopy, while the formation of phosgene decreased and the flow rate of carbon monoxide increased, converging to the set fed stream (Figure 3A).

**FIGURE 3:**
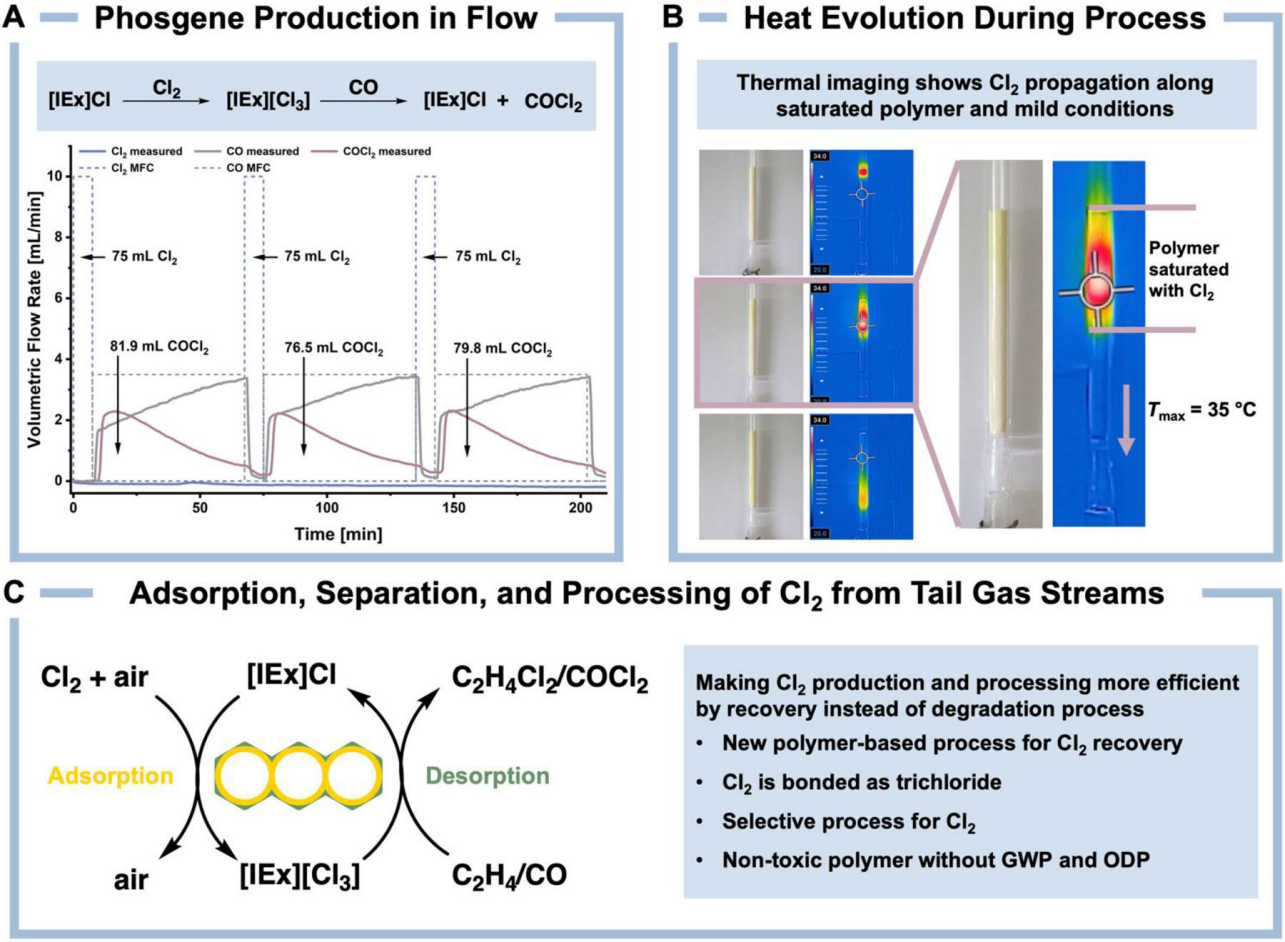
A) Conversion of adsorbed chlorine (Cl_2_) to phosgene (COCl_2_) over three cycles. The phosgene yield was calculated by determining the volume of formed phosgene by integrating the IR‐derived time‐dependent volumetric flow rate, while the volume of chlorine loaded onto the ion exchange resin was set using a mass flow controller. Blue: chlorine; grey: carbon monoxide (CO); red: phosgene. (B) Heat evolution during chlorine adsorption process analyzed using a thermal imaging camera revealing hotspot temperatures of 35°C. (C) Circular process combining separation of chlorine from chlorine‐containing tail gas stream, while generating the ion exchange resin trichloride ([IEx][Cl_3_]) (left) and chemical conversion of the adsorbed chlorine to 1,2‐dichloroethane (EDC) or phosgene, respectively, while regenerating the unloaded ion exchange resin ([IEx]Cl).

Besides its industrial applications, phosgene is also a valuable C1‐building block for the preparation of pharmaceuticals and agrochemicals [49]. As stable isotope‐labeled substrates are powerful tools to monitor drugs in biological systems and investigate metabolic processes, we telescoped the developed phosgene synthesis to a safe and simple batch procedure. Utilizing ^13^CO and [IEx][Cl_3_] as a chlorination agent, ^13^COCl_2_ could be prepared in 90% yield and easily be isolated both in pure form or as an easy‐to‐handle solution in toluene (Figure S5).

Finally, we investigated the exothermicity of the chlorine adsorption and conversion process. By using a non‐temperature‐controlled adsorber column, the chlorine adsorption process was analyzed with a thermal imaging camera. When a mixture of 10% chlorine in air was passed with a flow rate of 100 mL/min through the adsorber column, a moderate heat evolution reaching hotspots of only 35°C was observed (Figure 3B and Movie S1). Interestingly, the hotspot area traveled through the adsorber column, displaying a rapid local adsorption process and a continuously progressing saturation of the ion exchange resin. Accordingly, the conversion of a 5% mixture of ethylene in air to EDC with a flow rate of 95 mL/min on the adsorber column was elucidated. A similar heat profile was observed reaching a maximum temperature of 40°C on local hotspots (Figure S26 and Movie S2). These experiments demonstrate that the exothermicity of both processes can easily be controlled by ordinary cooling devices, facilitating an upscaling to industrial dimensions.

Based on the presented results, we assume that the use of our system for the recovery and upcycling of Cl_2_ from tail gas streams could increase the overall production efficiency of CAE significantly, which would come along with great potential savings (Supporting Information). First estimations suggest that for a typical Cl_2_ production plant with a production volume of 300 kt/a, 31,000 MWh of electrical energy could be saved every year reducing the CO_2_ foodprint of this plant by ∼13,000 t. Taking also the value of saved NaOH into account, this would add up to an annual saving of 3.11 million € by also addressing the Sustainable Development Goals (SDGs) 6, 9, 12, and 15 of the United Nations (UN).

In conclusion, we developed a circular process that combines separation and recycling of chlorine from industrially abundant chlorine‐containing tail gas streams utilizing a solid and commercially available ion exchange resin (Figure 3C). By contact with chlorine gas, the ion exchange resin of the type [IEx]Cl forms the corresponding trichloride [IEx][Cl_3_] as identified by Raman spectroscopy and in analogy to other known trichloride‐based systems. The chlorine adsorption by [IEx]Cl was found to be an exothermic and reversible process, allowing an efficient chlorine release by applying heat and reduced pressure. Flow experiments proved the fast and selective adsorption of chlorine from chlorine/air mixtures at various chlorine concentrations, temperatures, and flow rates, allowing a broad operational window. The adsorbed chlorine can be released almost quantitatively in high purity by applying heat and reduced pressure. Alternatively, the adsorber is regenerated by the direct conversion with ethylene to the base chemical 1,2‐dichloroethane or with carbon monoxide to phosgene. This enables the efficient recycling of chlorine from tail gas streams that account for global chlorine losses on a million‐ton scale and the direct upcycling of chlorine to valuable base chemicals in a safe combined process. Overall, the presented approach opens a promising perspective toward the economic valorization of tail gas streams and for a more circular and sustainable chemical industry.

## Author Contributions

**Gesa H. Dreyhsig**: conceptualization, investigation, writing – original draft, validation, visualization, methodology. **Merlin Kleoff**: supervision, writing – review and editing, funding acquisition, conceptualization. **Patrick Voßnacker**: formal analysis, visualization, validation, conceptualization, investigation, writing – review and editing. **Elisabeth Dorn**: investigation, methodology. **Jacob H. Richter**: methodology, investigation. **Sebastian Riedel**: writing – review and editing, project administration, supervision, funding acquisition.

## Conflicts of Interest

The authors declare the following competing financial interest: M. K., P. V., and S. R. are inventors on a patent pending related to this work (EP23206621, filed 2023). P. V., R. W., and S. R. are inventors on two patents pending related to this work (WO2022128951 A1, filed 2022 and WO2022128950 A1, filed 2022). The authors declare that they have no other competing interests.

## Supporting information



The authors have cited additional references within the Supporting Information [50, 51, 52, 53, 54, 55]. **Supporting File 1**: anie73820‐sup‐0001‐SuppMat.pdf.

**Supporting File 2**: anie73820‐sup‐0002‐Loading_Chlorine.mp4.

**Supporting File 3**: anie73820‐sup‐0003‐Release_Ethylene.mp4.

## Data Availability

The data that support the findings of this study are available from the corresponding author upon reasonable request.
