# Supplementary material for: Separation and Valorization of Chlorine From Tail Gas Streams
Source: Angew Chem Int Ed Engl. 2026 Aug 11;65(40):e3641857. doi: 10.1002/anie.3641857 (PMC13618281; doi:10.1002/anie.3641857)
Supplement: Supplementary file 1 [file ANIE-65-e3641857-s002.pdf]

Supporting Information for

Separation and Valorization of Chlorine from Tail Gas Streams

Gesa H. Dreyhsig,[a] Merlin Kleoff,[a] Patrick Voßnacker,[a] Elisabeth Dorn,[a] Jacob H. Richter,[a] Sebastian Riedel*[a]

Corresponding authors: s.riedel@fu-berlin.de

The PDF file includes:

Materials and Methods
Schemes S1 to S4
Figures S1 to S28
Tables S1
Movies S1 to S2
References

Table of Contents

Materials and Methods…………………………………………………………………….. 3

General Procedure for the Preparation of the Polymer [IEx]Cl…………………………. 3

Determination of Cl_2 Storage Capacities of [IEx]Cl………………………………………… 4

Determination of $C_2H_4Cl_2$ Storage Capacities of [IEx]Cl…………………………………… 5

Determination of $COCl_2$ Storage Capacities of [IEx]Cl……………………………………... 5

Determination of HCl Storage Capacities of [IEx]Cl……………………………………...... 6

Determination of Further Storage Capacities of [IEx]Cl…………………………………... 6

Batch Synthesis of Phosgene Using $[IEx][Cl_3]$…………………………………………… 6

Batch Synthesis of ^{13}C-labeled Phosgene Using $[IEx][Cl_3]$……………………………….. 7

Vapor Pressure Curves…………………………………………………………………… 8

Experimental Flow Setup…………………………………………………………………….. 8

Adsorption and Separation of Chlorine from Gas Streams Using [IEx]Cl……………… 11

Production of EDC by Unloading $[IEx][Cl_3]$ by Reacting it with Ethylene in Flow……… 12

Production of $COCl_2$ by Unloading $[IEx][Cl_3]$ by Reacting it with CO in Flow…………... 13

Thermal Release of Chlorine from the Adsorber Polymer………………………………… 13

Determination of Cycling Stability of Cl_2 Storage Capacities of [IEx]Cl………………… 13

Quantification of Reactants and Reaction Products in the Gas Streams……………….. 14

Raman Spectra………………………………………………………………………………… 17

IR Spectra……………………………………………………………………………………….. 18

Vapor Pressure Curves……………………………………………………………………… 19

Calibration Curves ……………………………………………………………………………. 20

Volumetric Flow Rates for the Flow Optimization for the Loading of [IEx]Cl…………… 25

Quantification of Chlorine Loading on $[IEx][Cl_3]$ …………………………………………… 27

Flow Rates for the Chlorine Release by Reacting $[IEx][Cl_3]$ with Ethylene in Flow……. 28

Flow Rates for the Chlorine Release by Reacting $[IEx][Cl_3]$ with CO in Flow…………… 30

Heat Evolution During Chlorine Loading and Chlorine Release………………………… 32

Calculation of Energy Consumption of European chlorine production………………… 34

Saving Potential for Chloralkali Electrolysis…………………………………………………. 35

Discussion of the Bonding Situation in $[IEx][Cl_3]$……………………………………………. 36

Movies…………………………………………………………………………………………… 37

References and Notes………………………………………………………………………… 38

Materials and Methods

Attention: all reactions were performed using gaseous and sometimes highly toxic substances under pressure, use appropriate safety precautions and handle with care.

All substances sensitive to water and oxygen were handled under an argon atmosphere using standard Schlenk techniques and a scroll pump vacuum up to 10^{-3} mbar. All gas capacity values of [IEx]Cl were corrected according to the gas volume of the used reaction vessel. All chemicals were obtained from commercial suppliers and were used without further purification if not stated otherwise. [IEx]OH and [IEx]Cl were dried in vacuo at 60 °C for 1 hour to 1 day prior to use, while all solvents were obtained anhydrous by storage over activated 3 Å molecular sieves. IR spectra were recorded using a Nicolet™ iS50 FT-IR spectrometer (Thermo Fisher Scientific). Eight scans with a resolution of 4 cm^{-1} were recorded. UV/Vis spectra were recorded with a Lambda 465 UV/Vis spectrometer (PerkinElmer Inc.). Raman spectra were recorded using the FT-Raman spectrometer MultiRAM (Bruker Scientific Instruments Division) with an excitation wavelength of 1064 nm (Nd:YAG laser). The spectrometer is equipped with a germanium detector, which was cooled with liquid nitrogen. All spectra were measured at room temperature, at 50–100 mW laser power and a spectral resolution of 4 cm^{-1}. OPUS 7.5 (Bruker Scientific Instruments Division) and OMNIC™ (Thermo Fisher Scientific) were used for evaluation of the recorded spectra, Origin2025 (OriginLab) for the graphical representation. In dependency to the used gas, different MFCs were used (GF080CXXC (air/Ar and Cl_2), SLA5850SC1AB1C2A1 (CO, C_2H_4), Brooks Instruments GmbH). All MFCs are connected via a Model 0254 power supply, readout and setpoint controller (Brooks Instruments GmbH) to a computer and are regulated using the 0250 Series Readout standalone LabVIEW™ application (Brooks Instruments GmbH). The heat evolution of the reaction within the adsorber column was recorded with a thermal imaging camera Flir model E96.

General Procedure for the Preparation of the Polymer [IEx]Cl

$$[IEx]OH \xrightarrow{\text{HCl (3.7 mol.\%)}} [IEx]Cl$$

Scheme S1 Anion exchange of [IEx]OH to [IEx]Cl using hydrochloric acid.

The ion exchange resin [IEx]OH is based on a crosslinked styrene-divinylbenzene co-polymer containing quaternary ammonium groups (**Scheme S1**). [IEx]OH (75 g) was washed with methanol (250 mL). After one hour, methanol was carefully decanted, before fresh methanol

(250 mL) was added. The suspension was left standing for twelve hours, the methanol carefully decanted once more, and the polymer dried in vacuo. Subsequently, the polymer’s hydroxide function was exchanged to a chloride anion by carefully adding hydrochloric acid (3.7 mol.%, 600 mL), before the polymer was washed with methanol (3x 75 mL), tetrahydrofuran (3x 125 mL), and dichloromethane (3x 125 mL). The colorless polymer [IEx]Cl was dried in vacuo at 60 °C for 24 hours.

The polymer was pre-chlorinated by carefully adding chlorine gas up to a total pressure of 1050 mbar. Afterwards, excessive chlorine was removed in vacuo to obtain the prepared polymer-based adsorber polymer [IEx]Cl (**Figure S4**).

[IEx]Cl (pre-chlorinated): Raman, $\tilde{\nu}$ = 3011 (s), 2941 (s), 2912 (s), 1614 (s), 1456 (m), 1456 (m), 1223 (m), 1192 (m), 1001 (w) 976 (w), 893 (w), 762 (w) 722 (w), 643 (w) cm^{-1}.

Determination of Cl_2 Storage Capacities of [IEx]Cl in Batch

$$[\mathrm{IEx}]\mathrm{Cl} \quad + \quad \mathrm{Cl_2} \quad \rightleftharpoons \quad [\mathrm{IEx}][\mathrm{Cl_3}]$$

Scheme S2 Reversible adsorption of Cl_2 by the pre-chlorinated polymer-based adsorber material [IEx]Cl to form the corresponding trichloride [IEx][Cl_3].

The pre-chlorinated polymer-based adsorber material [IEx]Cl (1.93 g) was placed into a pressure-stable RETTBERG flask and loaded with chlorine until the system retained a pressure of 1000 mbar (**Scheme S2**). The amount of stored chlorine was investigated by determining the mass difference between the unloaded [IEx]Cl and the corresponding trichloride [IEx][Cl_3] (Raman: **Figure S4**). To analyze the long-term stabilities, cycle tests were performed repeating loading and unloading of the adsorber material with Cl_2 (**Figure S1**).

[IEx][Cl_3]: Raman, $\tilde{\nu}$ = 3026 (m), 2964 (m), 2920 (m), 1611 (m), 1451 (w), 1223 (w), 1196 (vw) 976 (vw), 891 (vw), 758 (vw), 714 (vw), 643 (vw), 475 (vs), 286 (m) cm^{-1}.

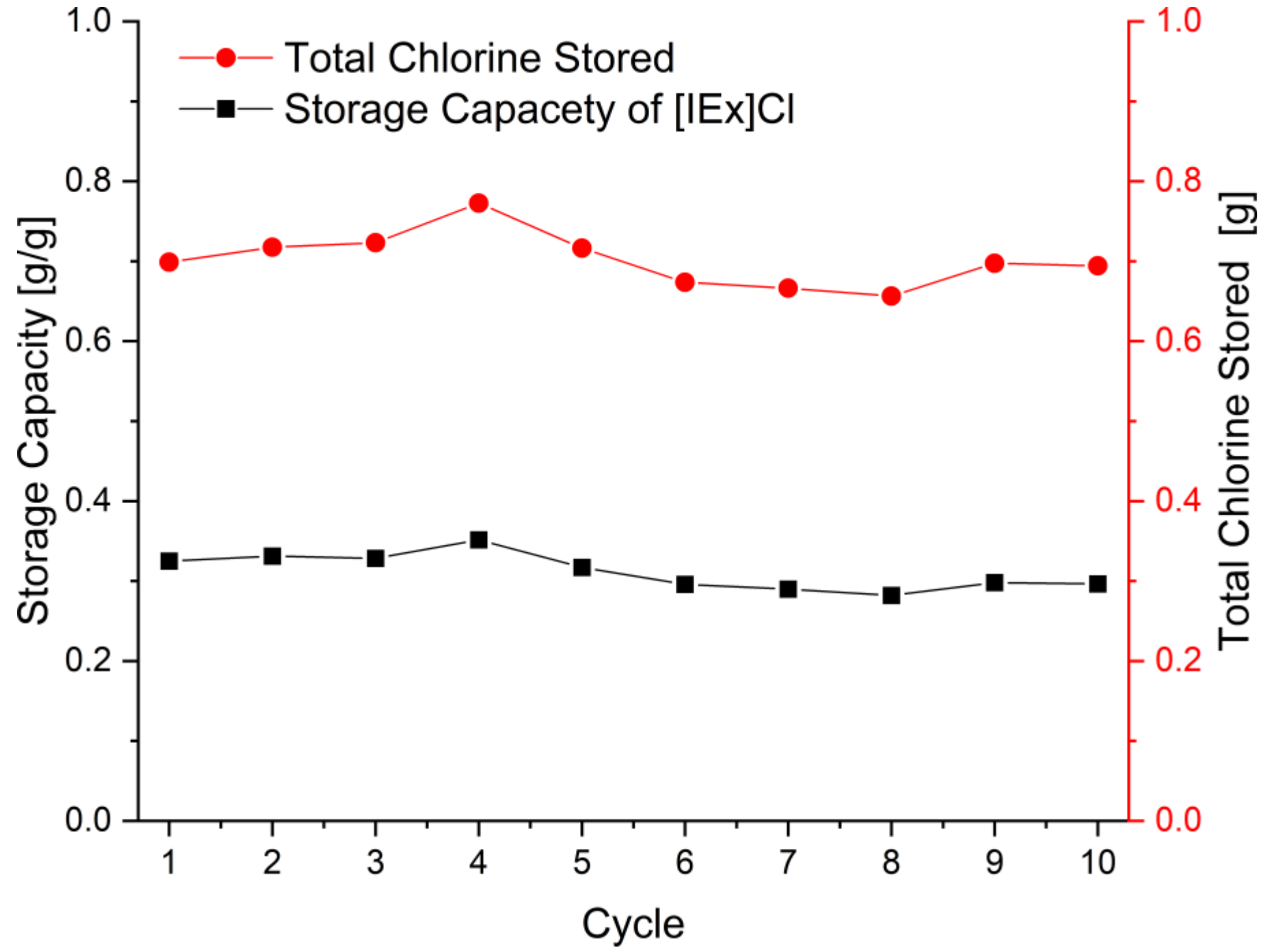

Figure S1 Storge capacities (black) and amount of total stored chlorine (Cl_2, red) on the adsorber material [IEx]Cl determined over 10 loading and unloading cycles.

Determination of $C_2H_4Cl_2$ Storage Capacities of [IEx]Cl

The pre-chlorinated polymer-based adsorber material [IEx]Cl (7.49 g) was placed into a pressure-stable RETTBERG flask and mixed with 1,2-dichloroethane (12.3 g, 125 mmol). The mixture was left standing for five hours before 1,2-dichloroethane was removed and the polymer dried in vacuo. The amount of adsorbed 1,2-dichloroethane (0.414 g, 4.18 mmol) was determined gravimetrically by the mass difference between the unloaded [IEx]Cl and the remained polymer to determine the storage capacity (0.0553 g g^{-1}).

Determination of $COCl_2$ Storage Capacities of [IEx]Cl

The pre-chlorinated polymer-based adsorber material [IEx]Cl (1.96 g) was placed into a 100 mL pressure-stable RETTBERG flask and mixed with phosgene (0.605 g, 6.16 mmol). The mixture was left standing for several hours before phosgene was removed and the polymer dried in vacuo resulting in the complete discharge of the polymer-based adsorber material, which agrees with all further observations.

Determination of HCl Storage Capacities of [IEx]Cl

The pre-chlorinated polymer-based adsorber material [IEx]Cl (2.06 g) was placed into a pressure-stable RETTBERG flask and loaded with hydrogen chloride until the system retained a pressure of 1000 mbar. The amount of stored HCl (0.472 g, 13.0 mmol, 0.222 g HCl/g [IEx]Cl) was investigated gravimetrically by determining the mass difference between the unloaded [IEx]Cl and the corresponding bichloride [IEx][HCl_2].

Determination of Further Storage Capacities of [IEx]Cl

The adsorption of 1-chlorbutane, chlorobenzene, 2-chloroaniline, and aniline by [IEx]Cl in the liquid phase was evaluated using quantitative NMR. For each tested compound, stock solutions were prepared in three molalities (0.400 mmol/g, 2.00 mmol/g, and 10.0 mmol/g). Since the required amounts of each compound vary with the amount of polymer used, 12 vials were filled with approximately 100 mg of the adsorber material. The polymer mass was used to calculate the amounts of compound required for each adsorption experiment. To create the stock solutions, the calculated amount was doubled and then dissolved in C_6D_6 (2 mL). Subsequently, the stock solutions were aliquoted. A smaller part was added directly to a standard NMR tube, while the other was added to one of the vials containing the polymer. Ferrocene (5.00 mg) in C_6D_6 as stock solution were added to the tubes. The remaining stock solutions of the adsorbate molecules were added to one vial each containing the polymer. The vials were capped, left, and stored for 24 h at room temperature. The liquid was then removed from the vials and transferred to NMR tubes. ^{1}H-QNMR experiments were conducted for all samples and references. The concentration of the solutions before and after exposure to the polymer was then determined from the total amount of adsorbate and the total volume of the liquid showing only the absorbance of 2-chloroaniline (40%) and aniline (75%).

Batch Synthesis of Phosgene Using [IEx][Cl_3]

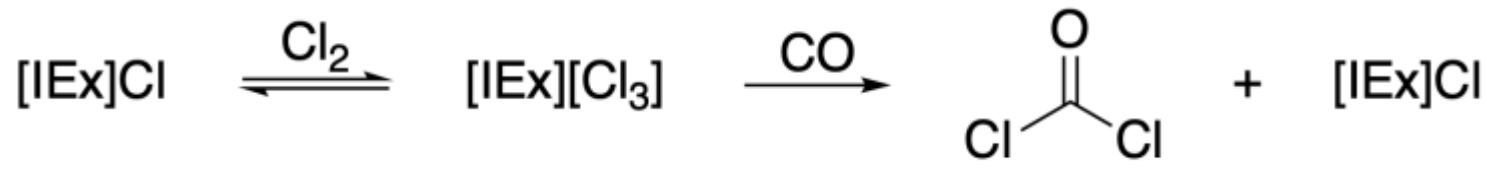

Scheme S3 Formation of [IEx][Cl_3] by the adsorption of Cl_2 by [IEx]Cl and its subsequent reaction with CO to phosgene.

Pre-chlorinated polymer-based adsorber material [IEx]Cl (1.38 g) was transferred to a 500 mL RETTBERG flask and loaded with chlorine until the system retained a pressure of

250 mbar (0.672 g, 9.48 mmol, 1.00 equiv.). Subsequently, carbon monoxide (550 mbar, 0.353 g, 12.6 mmol, 1.32 equiv.) was added to the flask (**Scheme S3**). The reaction mixture was covered with aluminum foil and left standing for 24 hours in the dark. Phosgene was obtained by distilling the volatile constituents in vacuo using cooling traps held at –130 °C ($COCl_2$) and –196 °C (HCl). All components were identified by its gas-phase IR spectra (**Figure S5**). The phosgene fraction was transferred into a pressure-resistant RETTBERG flask to determine the mass of phosgene gravimetrically (0.864 g, 8.74 mmol, **92%**). The spectroscopic data are consistent with those in the literature.[1] The polymer-based adsorber material can be reused after the reaction. After several synthesis cycles no more formation of HCl is observed and both cooling traps only contained $COCl_2$.

$COCl_2$: IR (ATR), $\tilde{\nu}$ = 1827 (vs), 1677 (w), 1411 (vw), 1008 (w), 851 (vs), 575 cm^{-1} (w).

Batch Synthesis of ^{13}C-labeled Phosgene Using [IEx][Cl3]

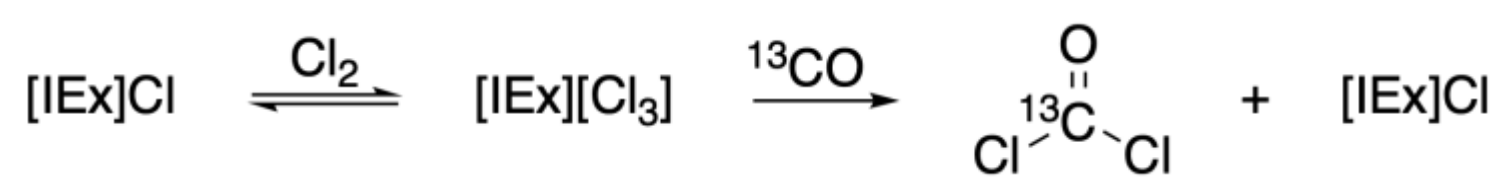

Scheme S4 Formation of [IEx][Cl_3] by the adsorption of Cl_2 by [IEx]Cl and its subsequent reaction with ^{13}CO to ^{13}C-labeled phosgene.

Pre-chlorinated polymer-based adsorber material [IEx]Cl (1.378 g) was transferred to a 500 mL RETTBERG flask and loaded with chlorine until the system retained a pressure of 250 mbar (0.649 g, 9.15 mmol, 1.00 equiv). Subsequently, ^{13}CO (550 mbar, 0.393 g, 13.6 mmol, 1.48 equiv) was added to the flask (**Scheme S4**). The reaction mixture was covered with aluminum foil and left standing for 24 hours in the dark. Phosgene was obtained by distilling the volatile constituents in vacuo using a cooling trap held at –196 °C. $^{13}COCl_2$ was identified by its gas-phase IR spectrum (**Figure S5**). The phosgene fraction was transferred into a pressure-resistant RETTBERG flask to determine the mass of $^{13}COCl_2$ gravimetrically (0.810 g, 8.19 mmol, **90%**). The spectroscopic data is consistent with these in the literature.[1] For easier handling a solution in toluene was generated by condensing the $^{13}COCl_2$ onto degassed toluene (8.2 mL, concentration of the resulting solution 1 mol/L)

$^{13}COCl_2$: IR (ATR), $\tilde{\nu}$ = 1784 (vs), 1623 (w), 1380(vw), 1005 (w), 826 (vs), 567 cm^{-1} (w).

Vapor Pressure Curves

4.26 g [IEx]Cl was transferred to a RETTBERG flask (118.5 mL) and treated with chlorine three times for pre-chlorination. After the third pre-chlorination cycle, the flask was heated to 20 °C and chlorine was gradually added to the [IEx]Cl in steps of 100 mbar. After the equilibration had been established the vapor pressure was recorded and the amount of Cl_2 was determined gravimetrically. Remaining Cl_2 in the gas phase was calculated using the ideal gas law and subtracted. After reaching an equilibrium vapor pressure of 1 bar, Cl_2 was removed under reduced pressure following the same procedure (**Figure S6**).

For the determination of the temperature dependent vapor pressure fully loaded [IEx][Cl_3] was filled into a glass autoclave connected to a digital manometer. The sealed vessel was heated stepwise (**Figure S7**). The temperature was held constant for about 60 min before the equilibrium vapor pressure was measured.

Experimental Flow Setup

A suitable flow setup was planned, constructed, and built to investigate adsorption, separation, and processing of chlorine from waste gas streams using polymer-based trichloride [IEx][Cl_3] (**Figure S2 and Figure S3**).

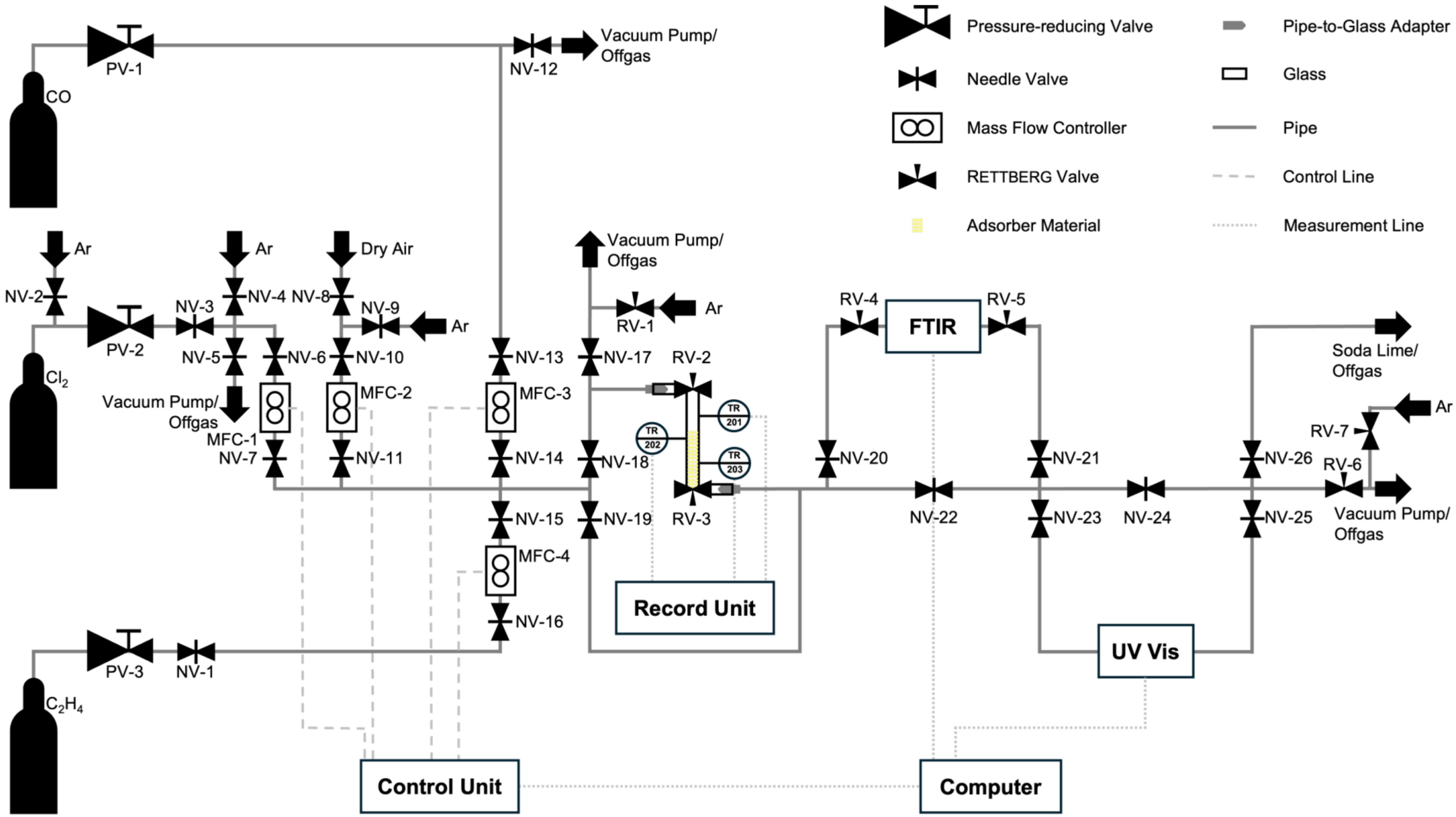

Figure S2. Process flow diagram of the experimental setup.

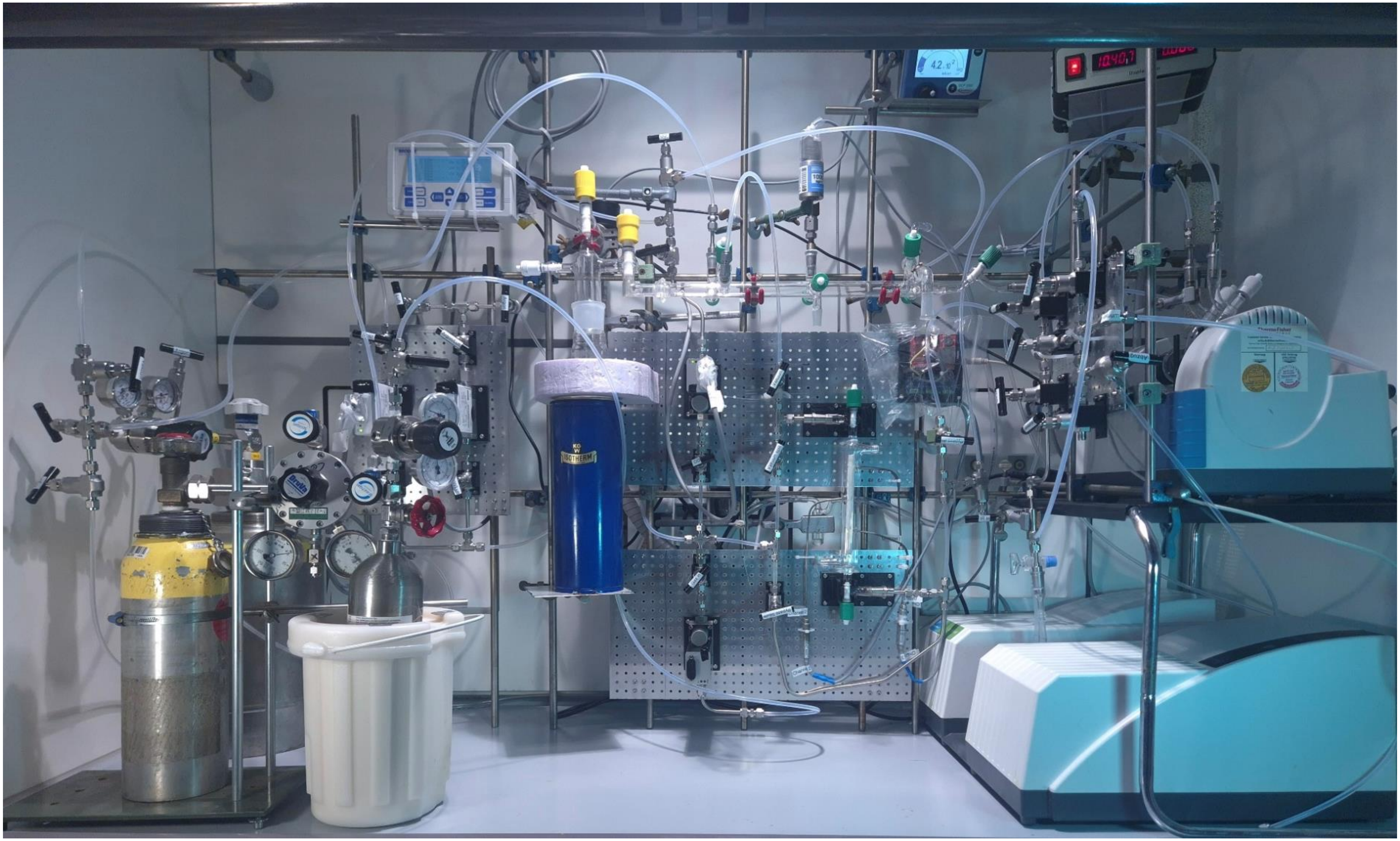

Figure S3. Photo of experimental setup.

The experimental flow setup has four dosing connection possibilities to different gases to produce gas mixtures of various components and compositions. The vapor pressures of carbon monoxide (CO), chlorine (Cl_2), and ethylene (C_2H_4) are down regulated using pressure-reducing valves PV-1 (CO), PV-2 (Cl_2), and PV-3 (C_2H_4). C_2H_4 is also connected via needle valve NV-1. PV-2 can be flushed with argon (Ar) via NV-2 and can be separated from mass flow controller MFC-1 by NV-3. MFC-1 regulates the Cl_2 mass flow, is isolated by NV-6 and NV-7 and can be flushed with Ar using NV-4. The part between NV-3, NV-4, and NV-6 can be evacuated via NV-5. Dry air or Ar can be dosed with MFC-2, which is isolated by NV-10 and NV-11, using NV-8 (dry air) and NV-9 (Ar). MFC-3 controls the gas flow of CO and is isolated by NV-13 and NV-14. The part between PV-1 and NV-13 can be evacuated via NV-12. NV-15 and NV-16 border MFC-4 dosing C_2H_4. NV-7, NV-11, NV-14, and NV-15 are connected to a pipe ending in a T-fitting to NV-18 and NV-19. NV-18 is connected to the glass column filled with the adsorber material and isolated via RETTBERG-Valves RV-2 and RV-3, and NV-17, which is used to evacuate the column or to flush it with Ar via RV-1. The column contains a fritted glass, is filled with the polymer (4.96 g), and connected to pipes with pipe-to-glass adapters. The glass-made column contains inlets for temperature sensors (TR201-TR203) after 2, 7 and 12 cm starting from the top of the column, to measure the temperature of the polymer. The data is logged using an external control unit (*Arduino Uno*, Arduino®, Monza, Italy). NV-19 enables the formation of a by-pass of the glass column to go directly to the analytical part of the plant. The analytical part consists of a central pipe from which the analytical devices branch off by means of T-pieces. NV-22 and NV24 enables division of the central pipe in different segments. IR spectra can be measured using a FTIR spectrometer. The IR cell is bordered via RV-4 and RV-5 and separated from the plant by NV-20 and NV-21. UV/Vis spectra are recorded with a UV/Vis spectrometer and can be excluded from the plant with NV-23 and NV-25. RV-6 connects the plant with a vacuum pump for potential evacuation, whereas RV-7 can be used to flush the setup with Ar. NV-26 enables dispersion of the gas flow to off gas over a soda lime for neutralizing reactive exhaust gases like phosgene ($COCl_2$).

Adsorption and Separation of Chlorine from Gas Streams Using [IEx]Cl

All valves were closed. The experimental setup was evacuated by opening the needle valves NV-17 to NV-25 and the RETTBERG-Valves RV-2 to RV-6. Afterwards, RV-6 as well as NV-19 to NV-21 and NV-24 were closed. The system was filled with Ar before also closing NV-17 and RV-1 or RV-6 and RV-7. Subsequently, NV-3, NV-6 to NV-8, NV-10, NV-11 and NV-26 were opened. PV-

2 was used to reduce the Cl_2 pressure of the gas bottle to 2.5 bar. The mass flow controllers MFC-1 and MFC-2 were used to regulate the Cl_2 and the dry air mass flows, respectively. The polymer was fully loaded with Cl_2 by passing the mixed gas stream through the column. The formation of the polymer-based trichloride was indicated by a color-change from pale beige to yellow of the polymer. The reaction temperature was measured and logged (**Figure S25**) or controlled using a thermostat. UV/Vis spectra were recorded in a time interval of 30 s to verify the amount of Cl_2 in the gas stream (See **Figure S15** to **Figure S17** for the processed UV/Vis data). After the complete loading of the polymer, the gas streams were stopped by MFC-1 and MFC-2. RV-2 and RV-3 were carefully closed as well as PV-2, NV-3, NV-6 to NV-8, NV-10, NV-11 and NV-26. NV-19 was opened, and the setup was evacuated to remove all Cl_2 not bonded on the polymer by opening NV-17 and RV-6. All valves were closed. To avoid potential corrosion PV-2 can be flushed with Ar by opening NV-2, NV-3, and NV-5 while the Cl_2 bottle is closed. MFC-1 can be flushed using either the flushing setup of PV-2 with a closed NV-5 and opened NV-6, NV-7, NV-19, NV-22, NV-24, and NV-26 or by opening NV-4 instead of NV-3.

Production of EDC by Unloading [IEx][Cl_3] by Reacting it with Ethylene in Flow

All valves were closed. The experimental setup was evacuated by opening NV-17 to NV-24 and RV-4 to RV-6. Afterwards, RV-6 as well as NV-22 and NV-24 were closed. The system was filled with Ar before also closing NV-17, NV-19 and RV-1. Subsequently, NV-8, NV-10, NV-11, NV-15, NV-16, and NV-26 were opened. PV-3 and NV-1 were used to reduce and regulate the C_2H_4 pressure of the gas bottle. RV-2 and RV-3 were carefully opened. MFC-2 and MFC-4 were used to regulate the dry air, and the ethylene mass flows, respectively. EDC was produced by passing the C_2H_4-containing gas stream through the column containing the polymer-based trichloride. The reaction temperature was measured and logged (**Figure S26**) or controlled using a thermostat. IR spectra were recorded in a time interval of 30 s to verify the amount of C_2H_4 and produced EDC in the gas stream. UV/Vis spectra were recorded in a time interval of 30 s to check the amount of unreacted and released Cl_2 (**Figure S20** and **Figure S21** for the processed IR and UV/Vis data). After the completed reaction, PV-3, NV-8, NV-10, NV-11, NV-15, and NV-16 were closed. NV-19 was opened, and the setup was evacuated by opening NV-17 and RV-6. All valves were closed. In between runs the adsorber column was evacuated over night by opening NV-17 and RV-2 and closing NV-18 and RV-3 while the rest of the setup was flushed with a slow argon stream regulated by MFC-1 to avoid corrosion.

Production of $COCl_2$ by Unloading [IEx][Cl_3] by Reacting it with CO in Flow

All valves were closed. The experimental setup was evacuated by opening NV-17 to NV-25 and RV-4 to RV-6. Afterwards, RV-6 as well as NV-22 and NV-24 were closed. The system was filled with Ar before also closing NV-17, NV-19 and RV-1. Subsequently, NV-8, NV-10, NV-11, NV-13, NV-14, and NV-26 were opened. PV-1 was used to reduce to CO pressure of the gas bottle. RV-2 and RV-3 were carefully opened. MFC-2 and MFC-3 were used to regulate the dry air, and the CO mass flows, respectively. Phosgene was produced by passing the CO-containing gas stream through the column containing the polymer-based trichloride. The reaction temperature was controlled using a thermostat. IR spectra were recorded in a time interval of 30 s to verify the amount of CO and produced phosgene in the gas stream. UV/Vis spectra were recorded in a time interval of 30 s to check the amount of unreacted and released Cl_2 (**Figure S22** to **Figure S24** for the processed IR and UV/Vis data). After the completed reaction, PV-1, NV-8, NV-10, NV-11, NV-13, NV-14 and NV-26 were closed. NV-19 was opened, and the setup was evacuated by opening NV-17 and RV-6. In between runs the adsorber column was evacuated over night by opening NV-17 and RV-2 and closing NV-18 and RV-3 while the rest of the setup was flushed with a slow argon stream regulated by MFC-1 to avoid corrosion.

Thermal Release of Chlorine from the Adsorber Polymer

The ion exchange resin [IEx]Cl within the column was loaded with chlorine as described above (15 mL/min Cl_2, 135 ml/min air, 20 °C) and the amount of chlorine stored on the polymer adsorber was determined as described below (401 mL, 16.4 mmol, 1.17 g; 0.236 g Cl_2/g of [IEx]Cl). The column was subsequently connected to a colling trap cooled to –196 °C. The polymer column was heated to 80 °C (the thermostat was preheated to 80 °C to minimize heating time) and the stored chlorine was removed *in vacuo for* 1 hour. The amount of released chlorine was determined gravimetrically (0.826 g, 11.6 mmol) corresponding to a release of 71% (**Figure S18**).

Determination of Cycling Stability of Cl_2 Storage Capacities of [IEx]Cl

To investigate the cycling stability of the ion exchange resin [IEx]Cl over several adsorption and desorption cycles, the Cl_2 storage capacity of [IEx]Cl was determined as described below at the first adsorption cycle (480 mL, 19.7 mmol, 1.40 g, 0.282 g Cl_2/g of [IEx]Cl) and after its use over a time of 6 months (36 cycles; 442 mL, 18.1 mmol, 1.29 g, 0.259 g Cl_2/g of [IEx]Cl) at different reaction conditions showing a decrease of 8% (**Figure S19**).

Quantification of Reactants and Reaction Products in the Gas Streams

To determine the concentration of the reactants and reaction products after passing through the column filled with the polymer the gas stream was characterized by UV/Vis and IR spectroscopy. According to the Beer–Lambert law, the integrated band absorbance is proportional to the concentration of the absorbing species and thus to its partial pressure in the gas phase.

$$p_X \sim \int_{\lambda_1}^{\lambda_2} A_X(\lambda) d\lambda \quad (1)$$

Assuming constant optical path length and temperature the correlation between partial pressure and the integrated band absorbance can be written as:

$$p_X = k_x * \int_{\lambda_1}^{\lambda_2} A_X(\lambda) d\lambda \quad (2)$$

k_X was determined for the respective species by measuring calibration curves. For Cl_2, CO and C_2H_4 calibration curves were obtained by preparing gas mixtures with different concentrations using mass flow controllers (MFCs). Since the system is open to the atmosphere, the partial pressures of the individual gases were calculated from the volumetric flow rates set by the MFCs according to:

$$p_X = p_{atm} * \frac{\dot{V}_X}{\dot{V}_{tot}} \quad (3)$$

For the reaction products $C_2H_4Cl_2$ and $COCl_2$ by recording spectra of the neat substances at different total pressures.

The spectral regions used for integration were chosen to avoid detector saturation and overlap with adjacent absorption bands. For chlorine, only a partial section of the absorption band was integrated to prevent interference with the phosgene band. For CO and C_2H_4 a non-linear correlation between the integrated band absorbance and the partial pressure was observed. This behavior is likely related to the limited spectral resolution of the IR spectrometer, which does not fully resolve the rovibrational structure of the bands. A quadratic fit provided good agreement with the measured integrated band absorbances and was valid over a range of total volumetric flow rates. Therefore, a quadratic function was used for calibration.

$$p_{CO} = k_{CO} * \int_{\lambda_1}^{\lambda_2} A_X(\lambda)d\lambda + k_{2,\ CO} * \left(\int_{\lambda_1}^{\lambda_2} A_X(\lambda)d\lambda\right)^2 \quad (4)$$

The UV/Vis and IR spectra of the respective compounds with highlighted wavelength and wavenumber ranges used for integration are shown in **Figure S8** and **Figure S9**. A summary of all used integration ranges and determined proportionality constants is given in **Table S1** and the calibration curves are shown in **Figure S10** to **Figure S14**.

Table S1. Summary of determined proportionality constants

Substance	Spectroscopy	Integration range	k	k₂
Cl_2	UV	300 – 464 nm	1.29876	-
CO	IR	1995 – 2250 cm^{-1}	1.28583	0.07913
$COCl_2$	IR	1600 – 1730 cm^{-1}	2.95303	
C_2H_4	IR	1803 – 1962 cm^{-1}	8.99298	0.29339
$C_2H_4Cl_2$	IR	1178 – 1268 cm^{-1}	2.91205	

To quantify the total volumes of the gases passing through after interaction with the column material, the volumetric flow rates of the reactants and products were determined from the partial pressures obtained using the calibration curves.

The total volumetric flow rate of the reaction mixture can be calculated according to the:

$$\dot{V}_{tot} = \dot{V}_{Air} * \frac{p_{tot}}{p_{Air}} \quad (5)$$

Previous experiments showed that air does not interact with the polymer material. Therefore, air acts as an inert internal reference gas, allowing changes in the total volumetric flow rate caused by gas consumption or formation during the reaction to be quantified. The volumetric flow rate of air can be assumed to remain constant and equal to the setpoint value of the mass flow controller (MFC). Since the system is open to the atmosphere, the total pressure in the system is assumed to be equal to the atmospheric pressure. The partial pressure of air can therefore be calculated as

$$p_{Air} = p_{atm} - \sum p_{Reactants} - \sum p_{Reaction\ Products} \quad (6)$$

The volumetric flow rates of the individual compounds can then be calculated according to:

$$\dot{V}_X = \dot{V}_{tot} * \frac{p_X}{p_{tot}} \tag{7}$$

As the gas stream is characterized by IR and UV/Vis spectroscopy every 30 s, the volumetric flow rates of the individual species can be determined in a time-resolved manner. The total amount of compound X passing through the system is obtained by integrating the time-resolved volumetric flow rate over the reaction time:

$$V_X = \int_{t_1}^{t_2} \dot{V}_X(t) \tag{8}$$

To determine the amount of chlorine stored on the polymer, the volume of chlorine measured downstream of the column was subtracted from the total volume of chlorine introduced into the system, as defined by the set volumetric flow rate of the MFC.

$$V_{Cl_2;\, stored} = V_{Cl_2;\, MFC} - V_{Cl_2;\, \mathrm{measured}} \tag{9}$$

Raman Spectra

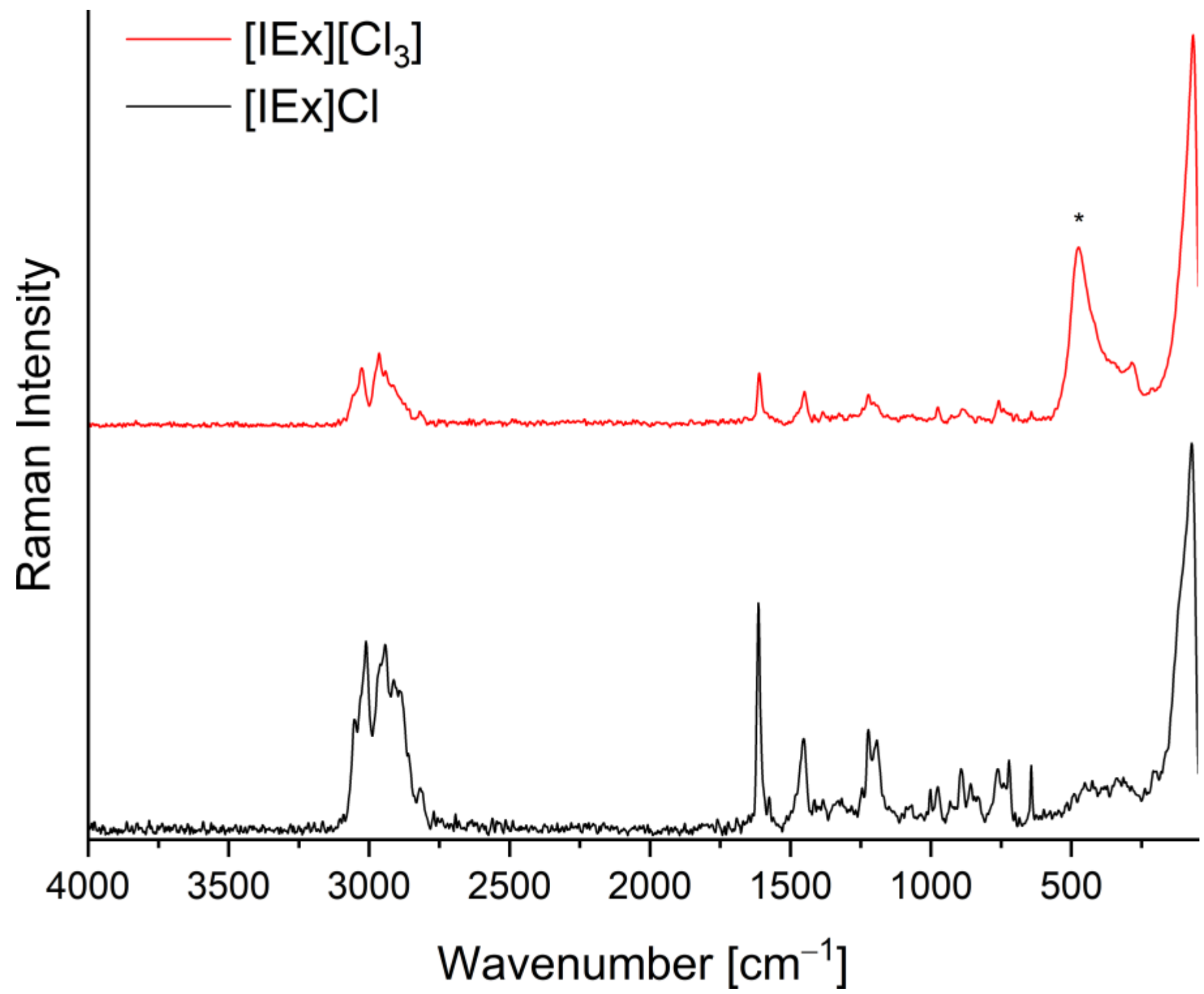

Figure S4. Raman spectrum of the [IEx]Cl (black) and [IEx][Cl_3] (red). The band highlighted with an asterisk corresponds to a trichloride species [Cl_3].

IR Spectra

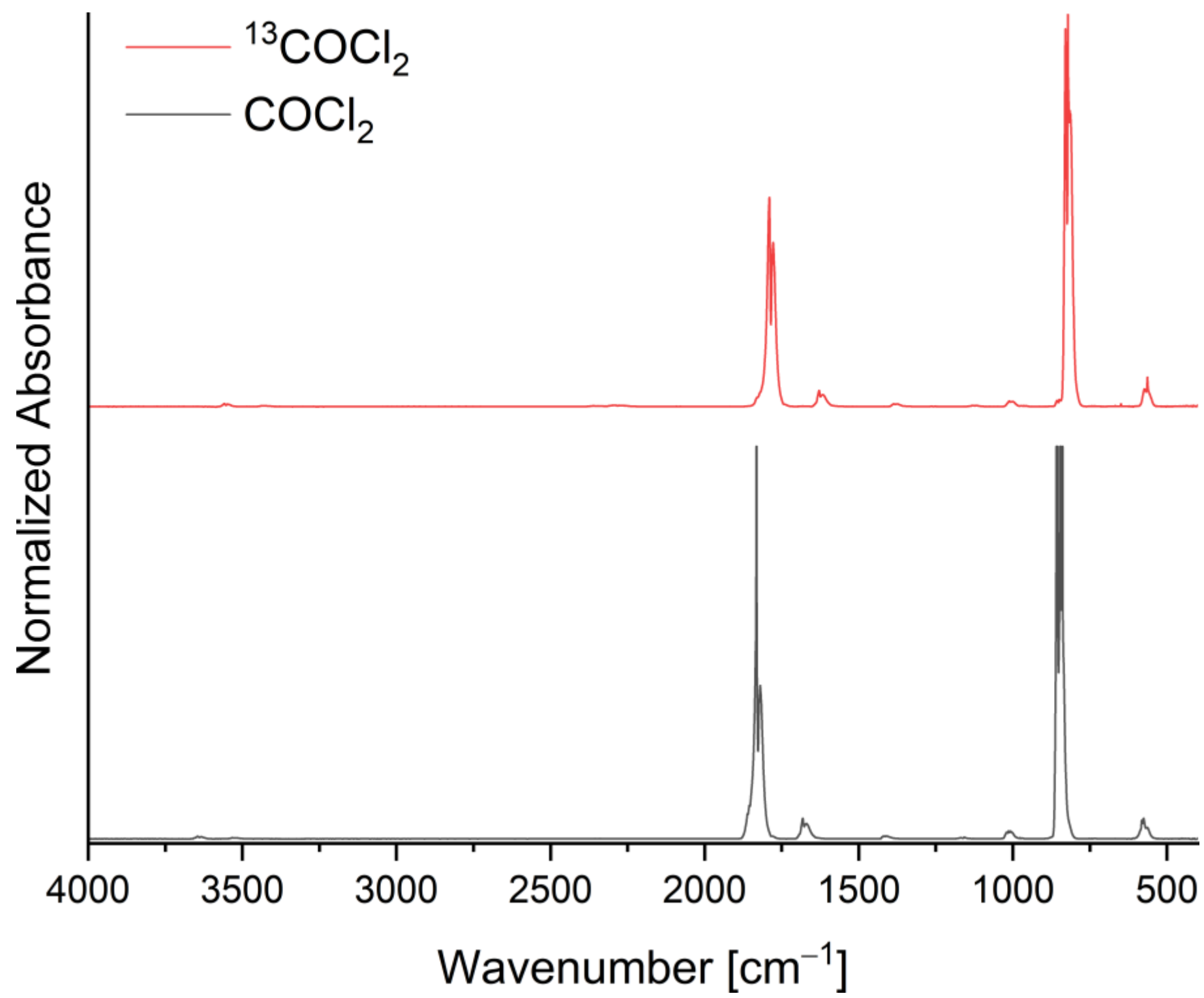

Figure S5. IR Spectrum of the purified reaction product ($COCl_2$) of the reaction of [IEx][Cl_3] with CO and ^{13}CO.

Vapor Pressure Curves

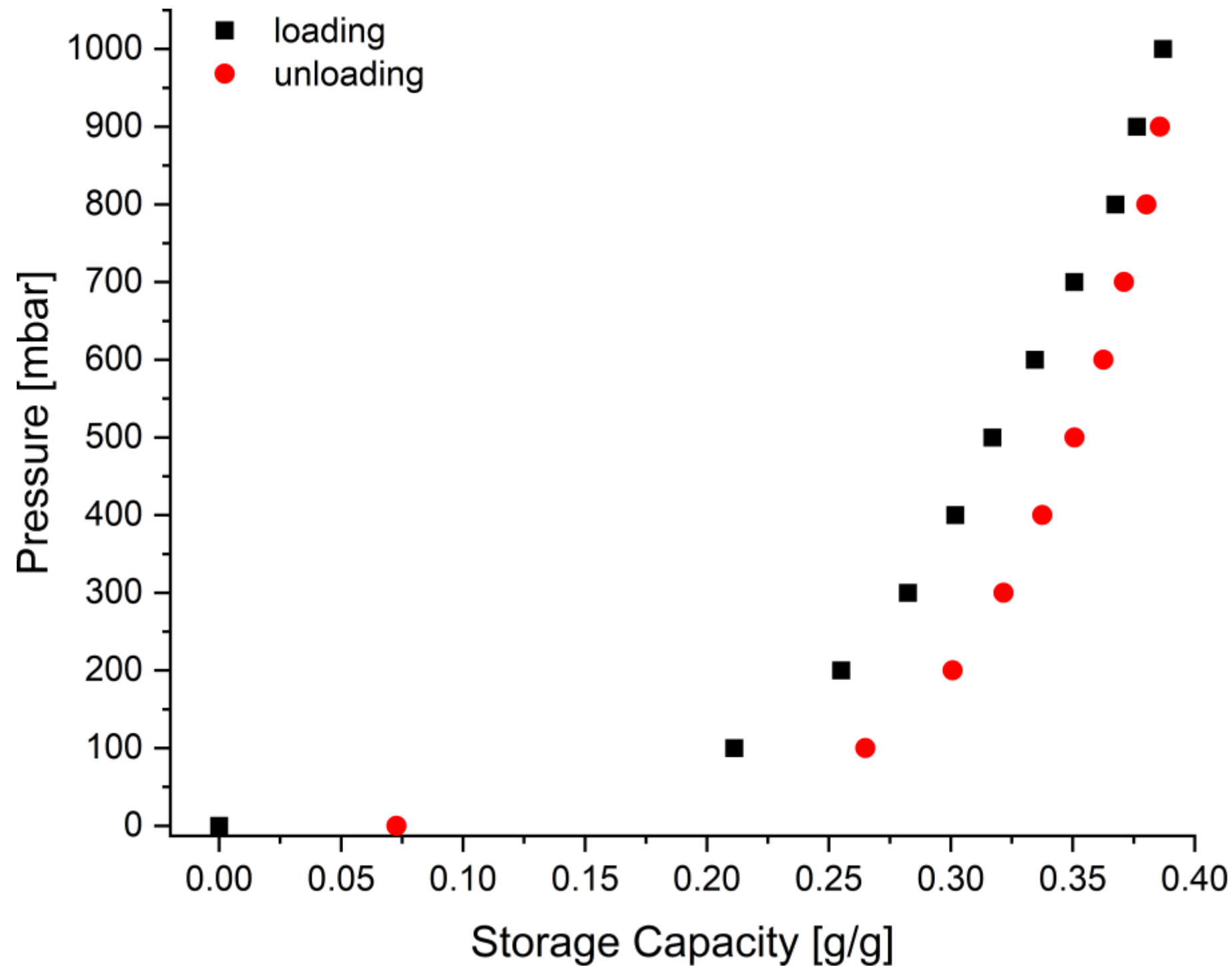

Figure S6. Storage capacities of [IEx][Cl_3] in dependence of the vapor pressure.

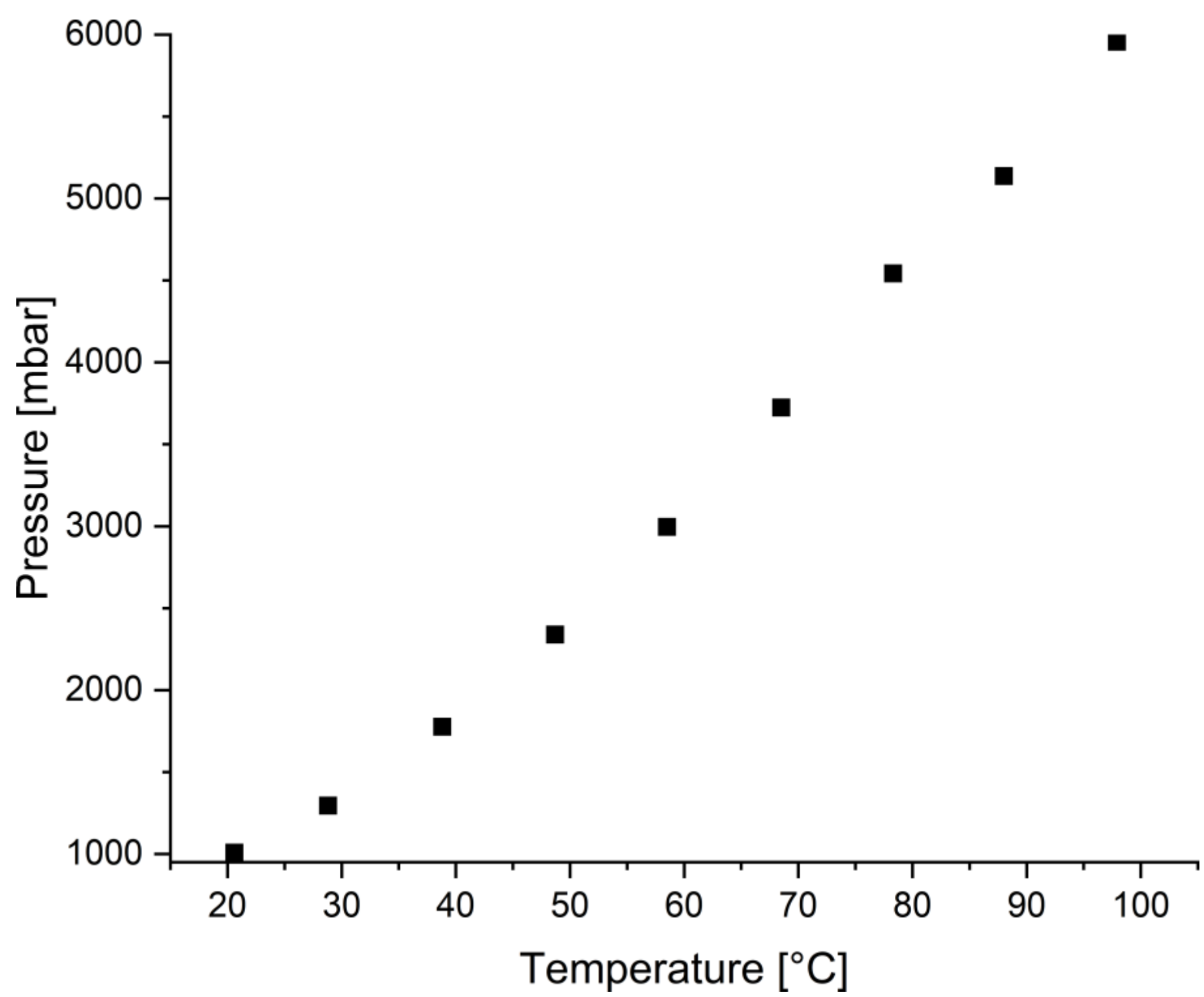

Figure S7. Temperature dependent vapor pressure curve of the fully loaded [IEx][Cl_3].

Calibration Curves

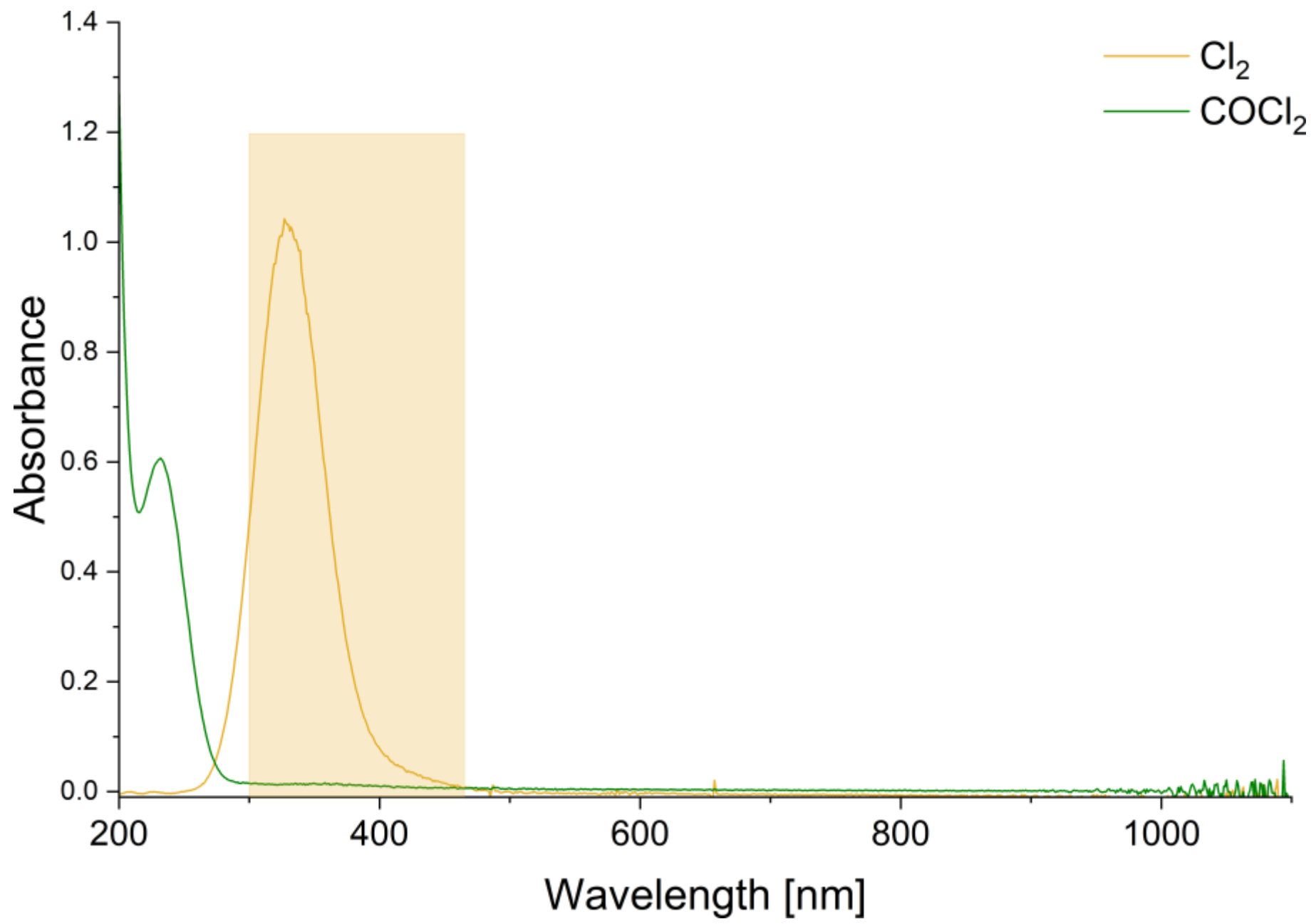

Figure S8. UV/Vis spectra of Cl_2 (orange) and $COCl_2$ (green) with highlighted wavelength area which was used for integration for the calibration curve.

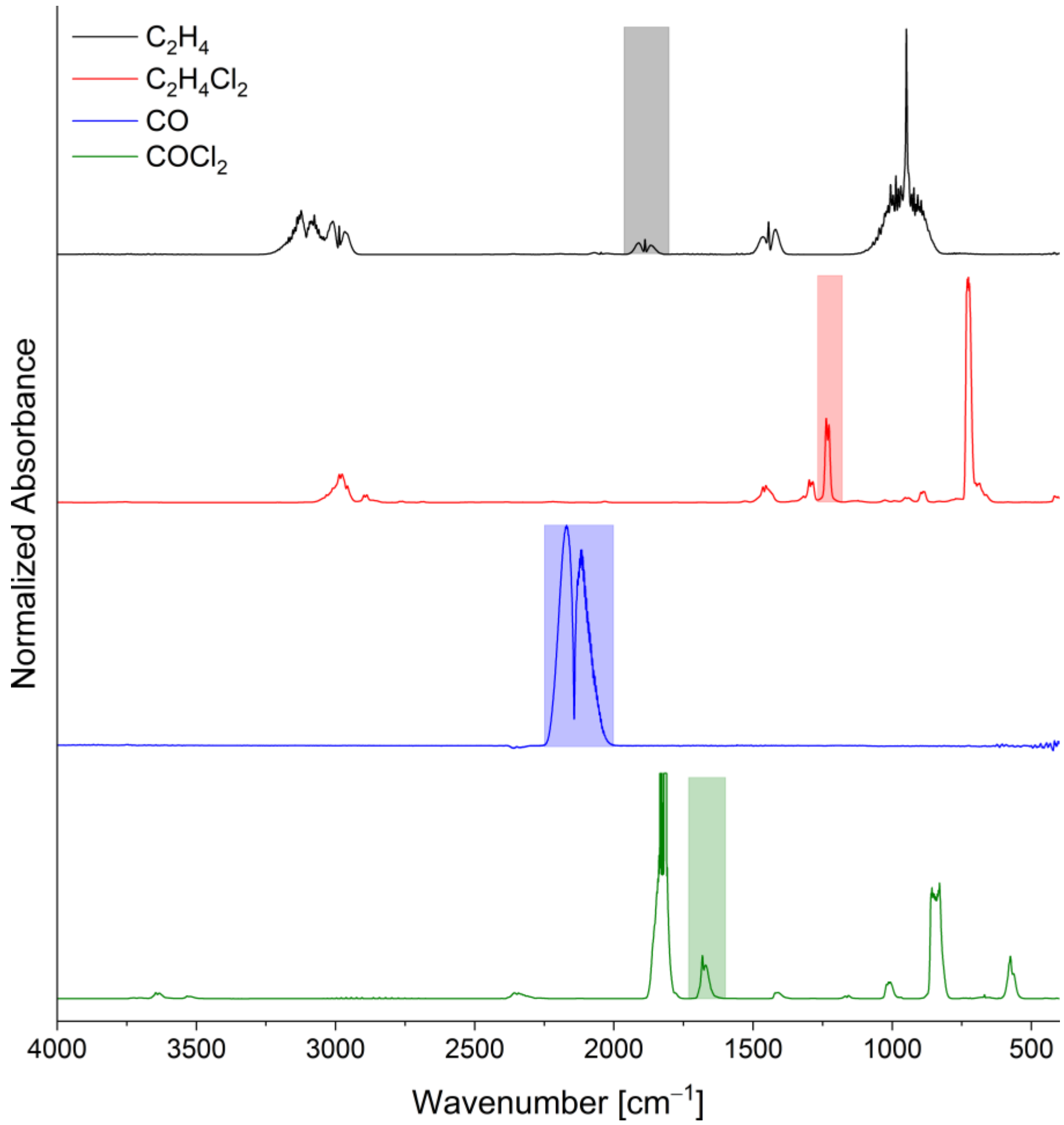

Figure S9. IR spectra of C_2H_4 (black), $C_2H_4Cl_2$ (red), CO (blue) and $COCl_2$ (green) with highlighted wavenumber areas which were used for integration for the calibration curve.

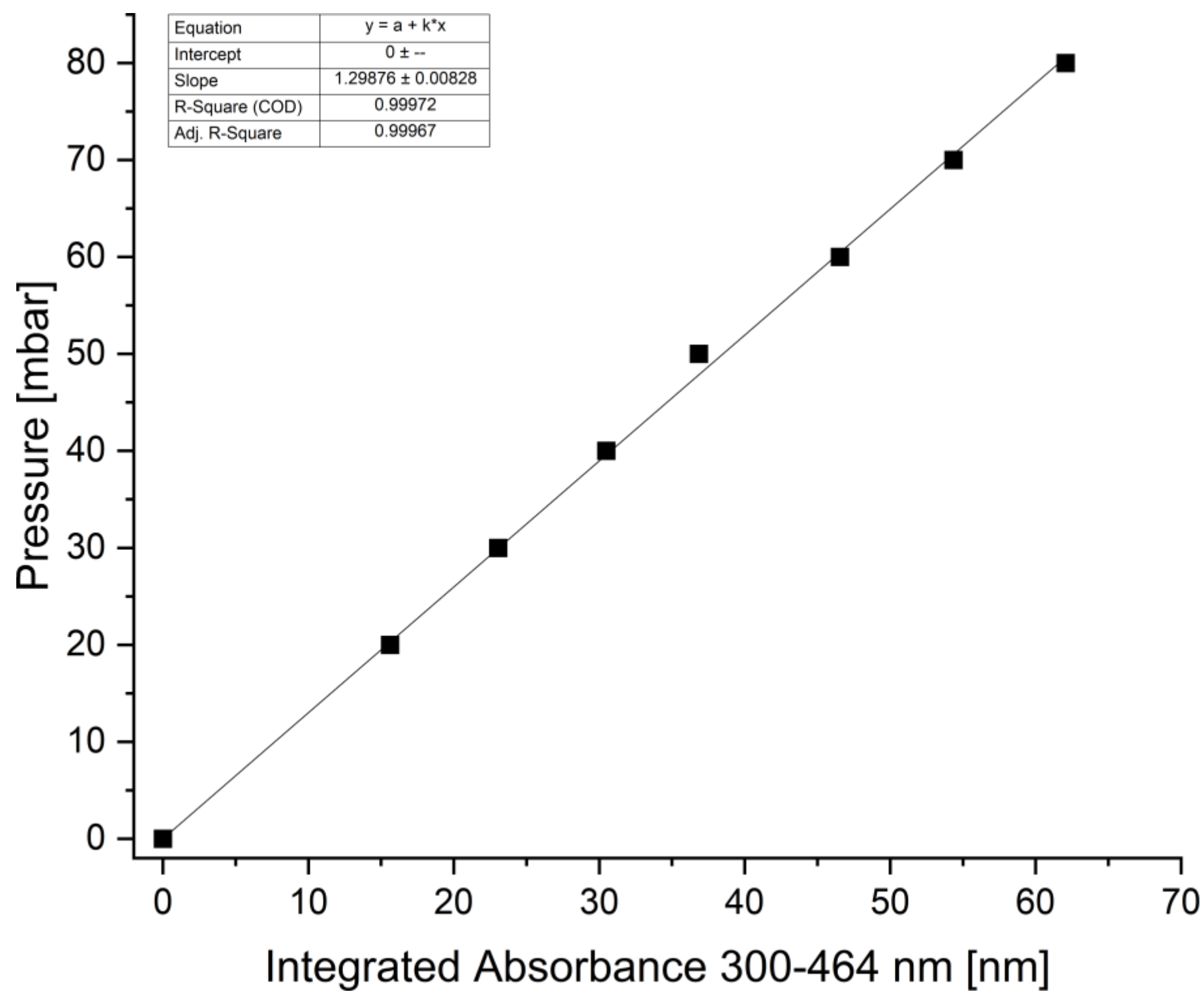

Figure S10. Calibration curve for Cl_2.

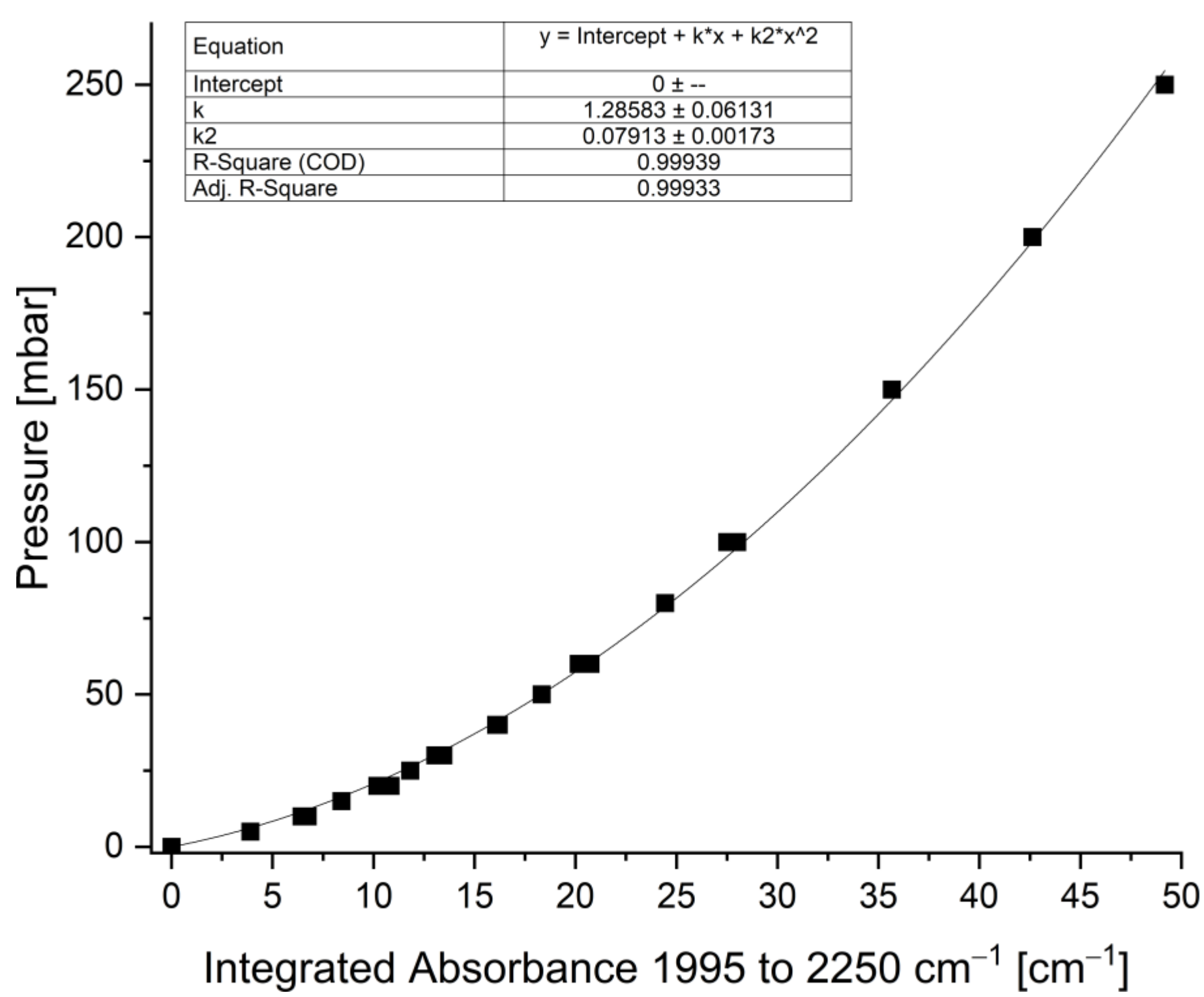

Figure S11. Calibration curve for CO.

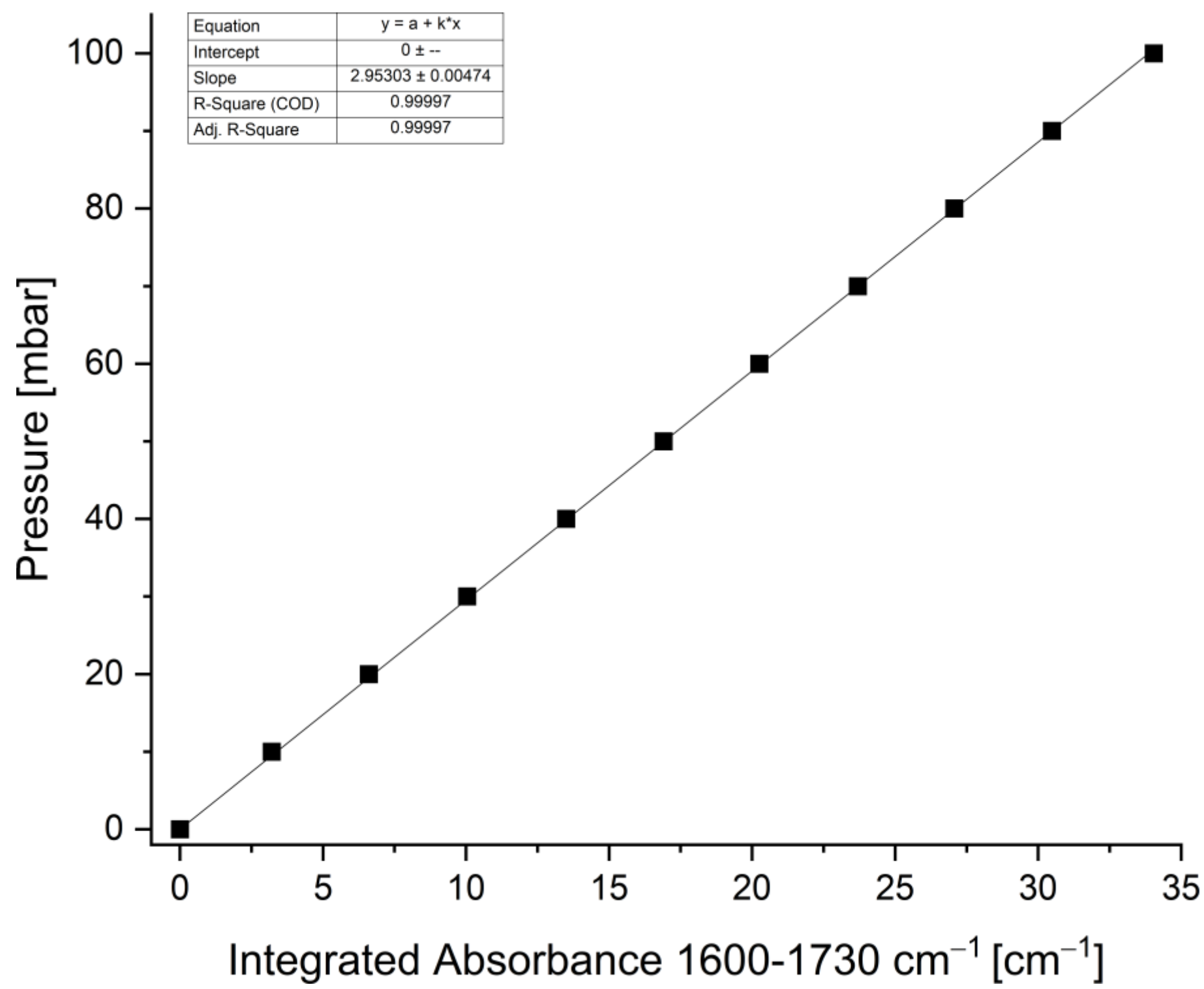

Figure S12. Calibration curve for $COCl_2$.

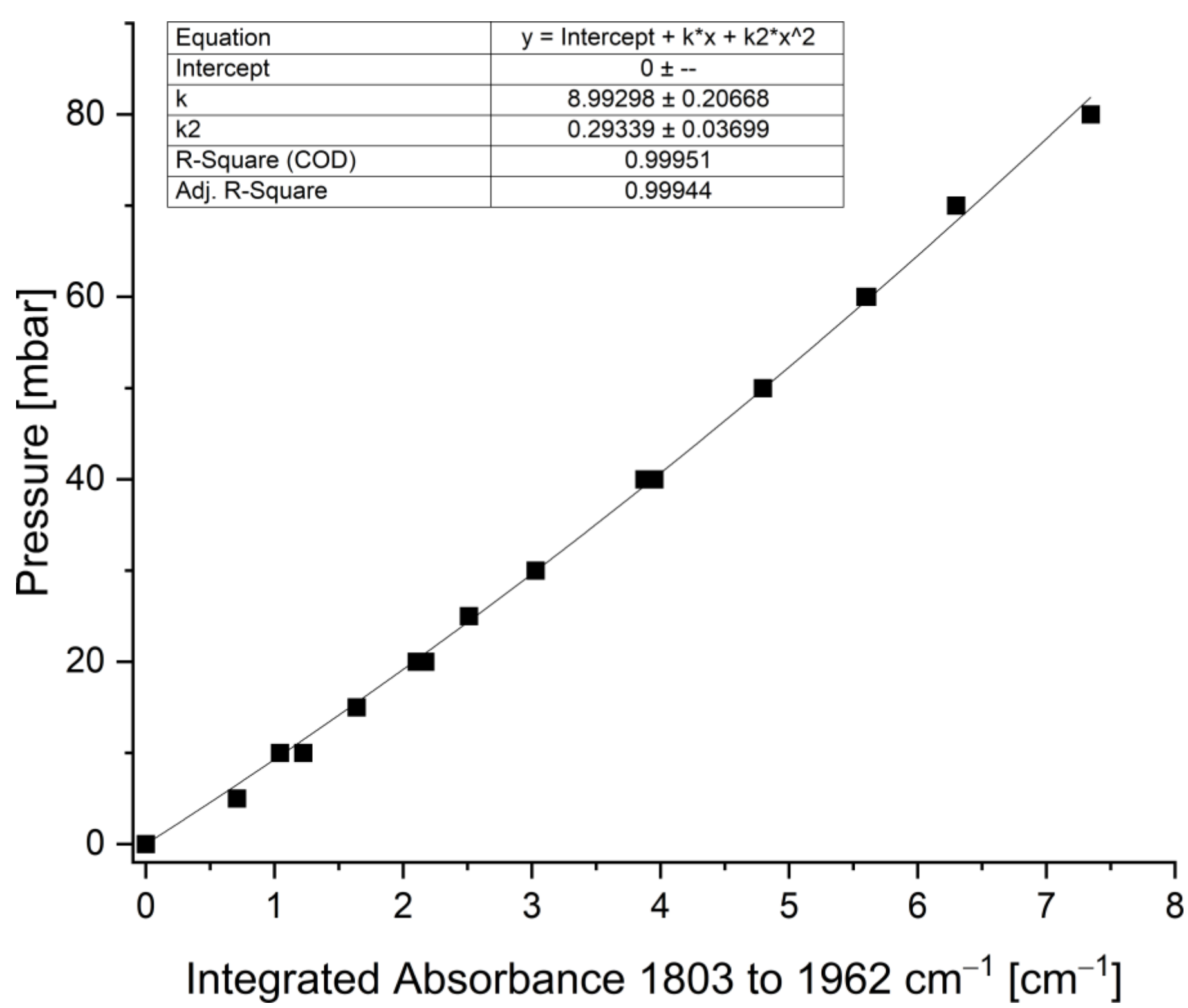

Figure S13. Calibration curve for C_2H_4.

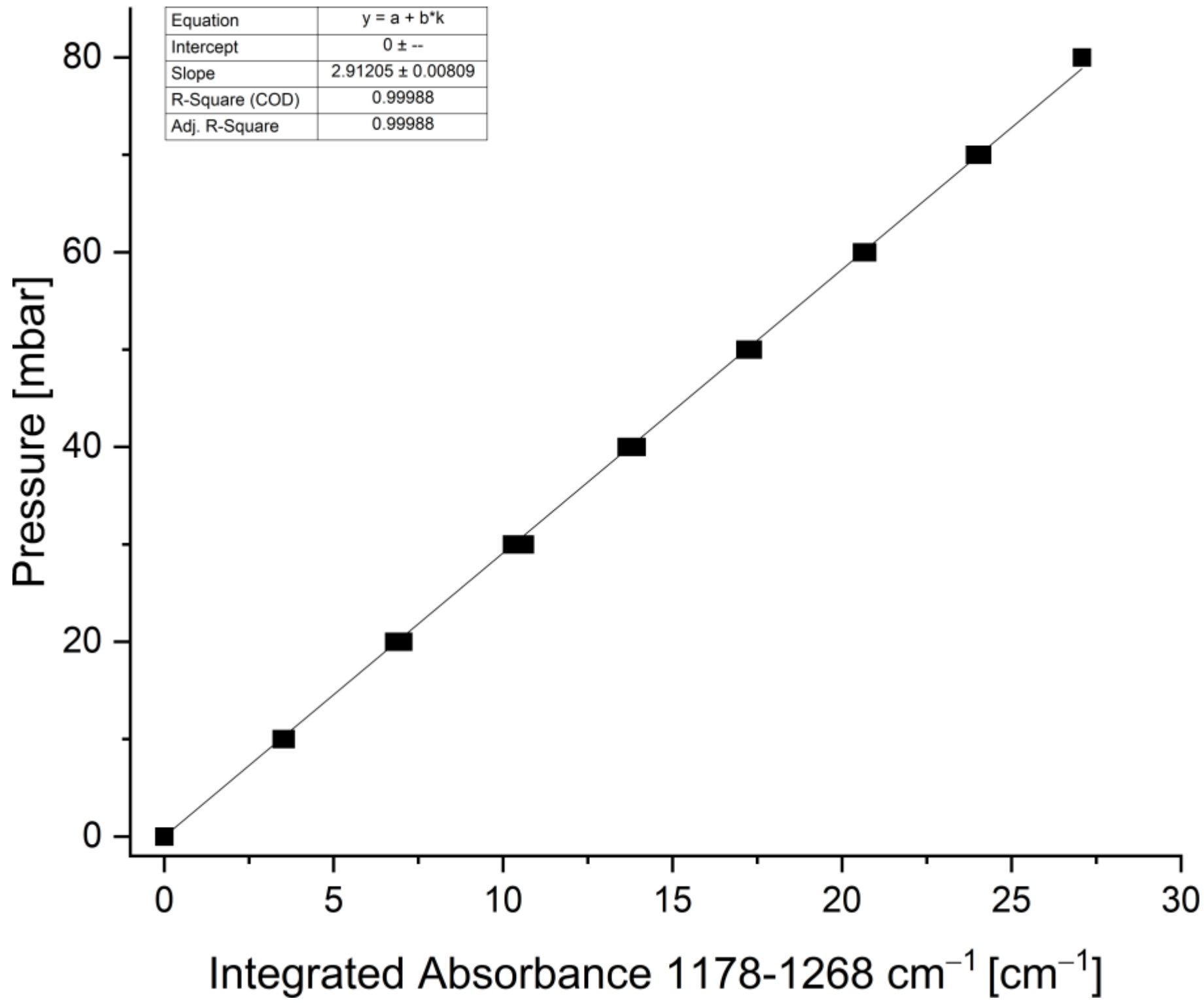

Figure S14. Calibration curve for $C_2H_4Cl_2$.

Volumetric Flow Rates for the Flow Optimization for the loading of [IEx]Cl

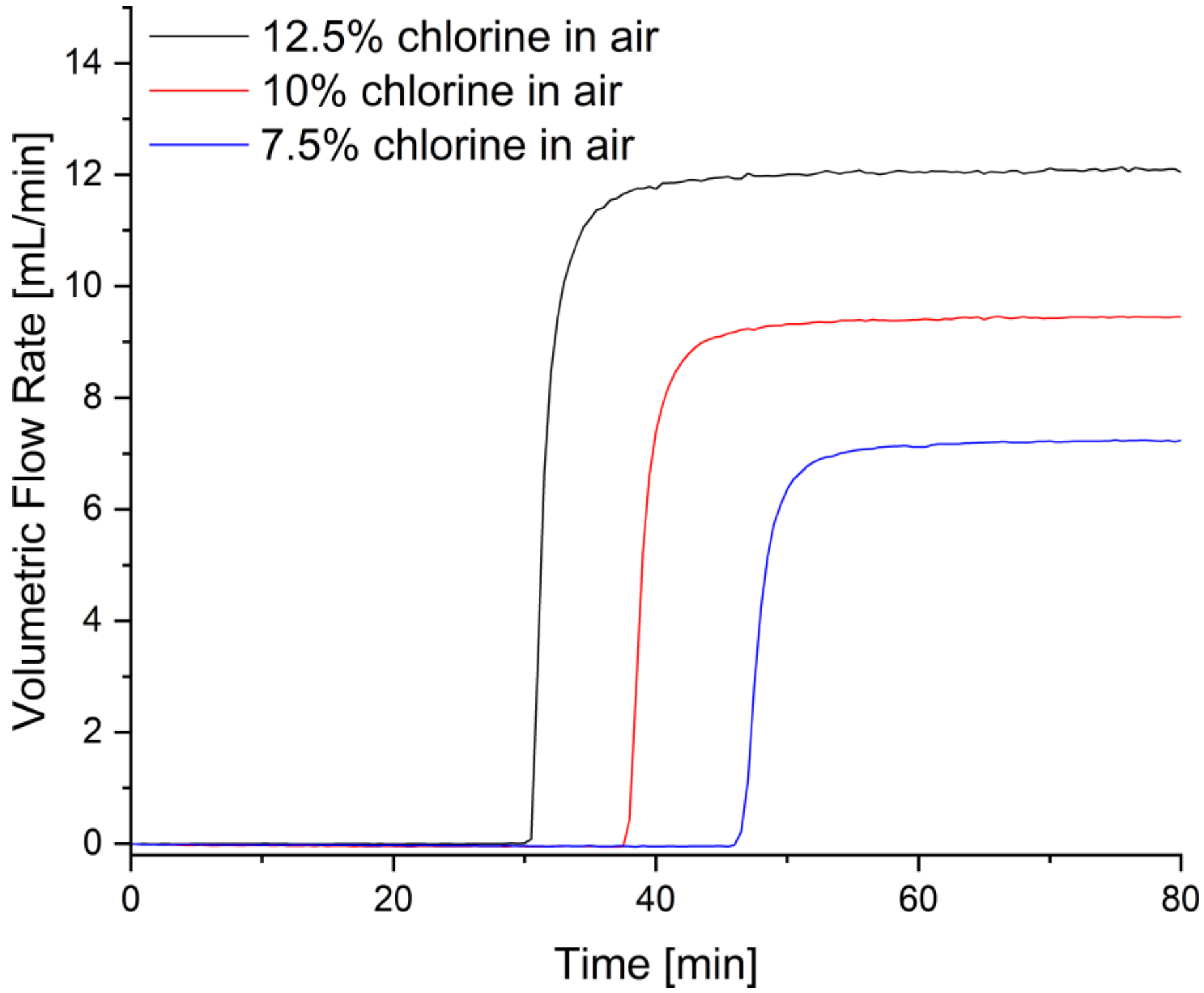

Figure S15. Comparison of the determined volumetric flow rates for the loading of [IEx]Cl at different chlorine concentrations with a constant total flow rate of 100 mL/min.

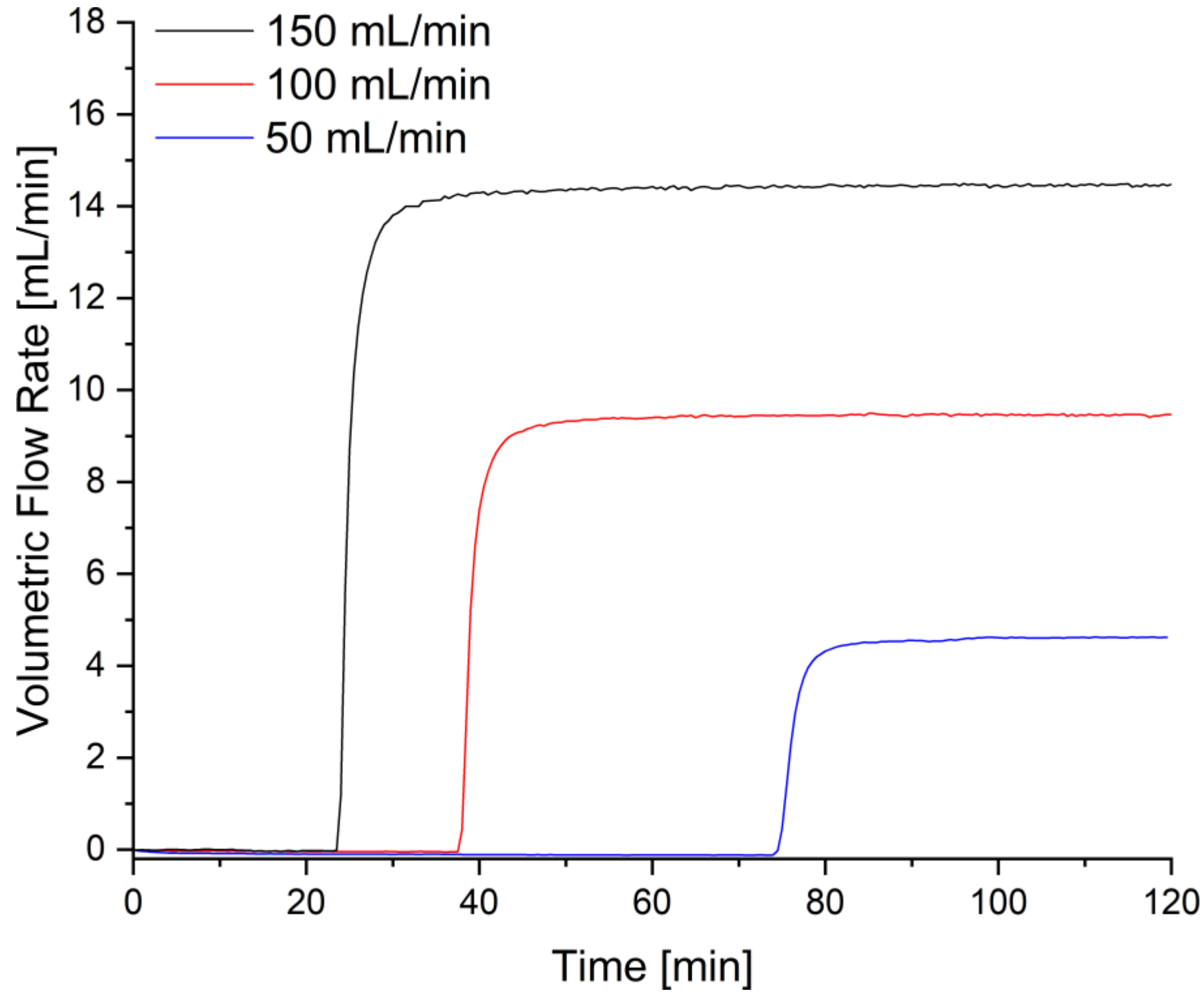

Figure S16. Comparison of the determined volumetric flow rates for the loading of [IEx]Cl with different total flow rates with a constant chlorine concentration of 10% and a constant temperature of 20 °C.

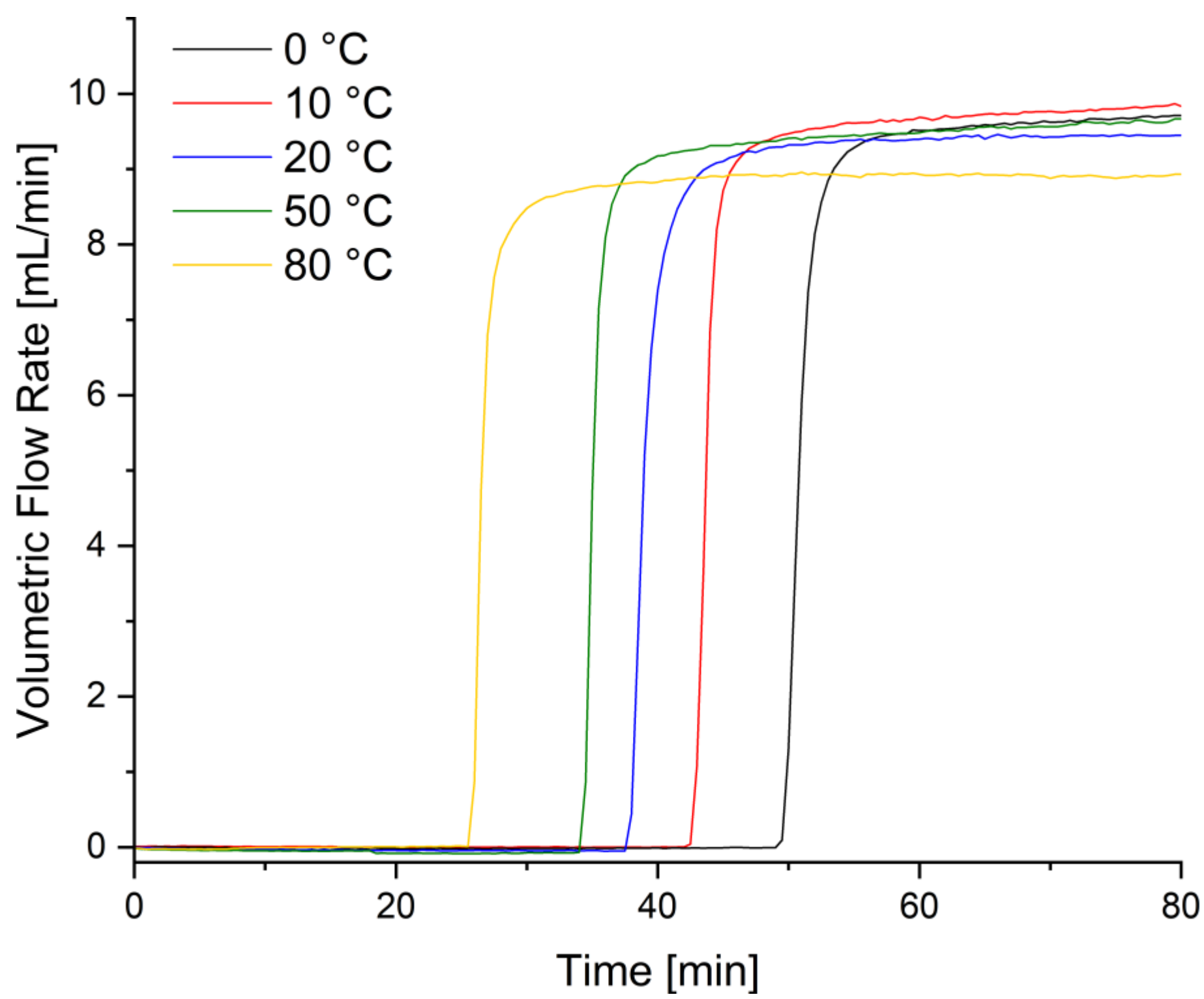

Figure S17. Comparison of the determined volumetric flow rates for the loading of [IEx]Cl at different temperatures with a constant chlorine concentration of 10% and a constant total flow rate of 100 mL/min.

Quantification of Chlorine Loading on [IEx][Cl3]

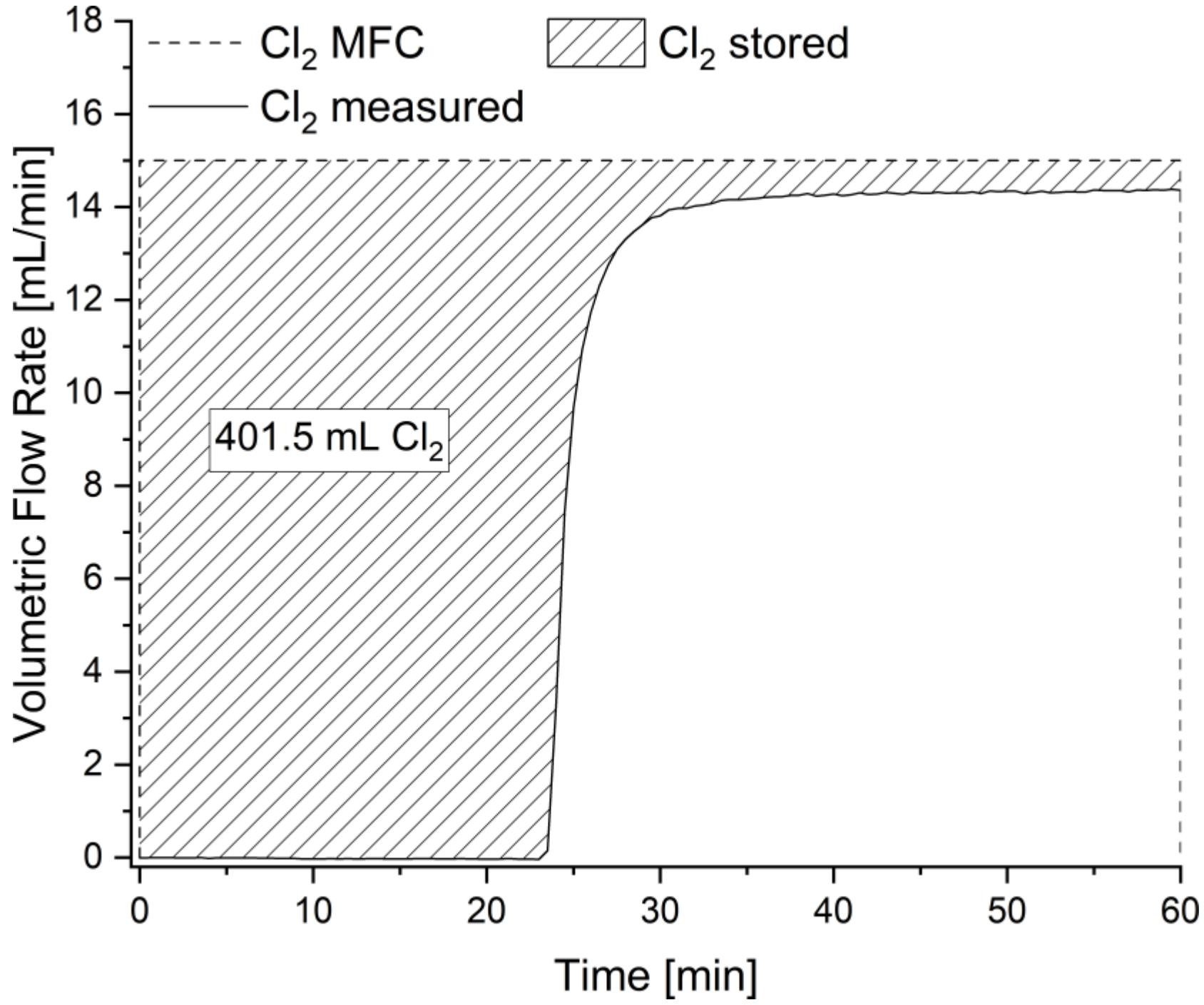

Figure S18. Loading of the polymer adsorber with 10% Cl_2 in air (total flow rate 150 ml/min, temperature 20 °C) and determination of the chlorine stored on the adsorber polymer.

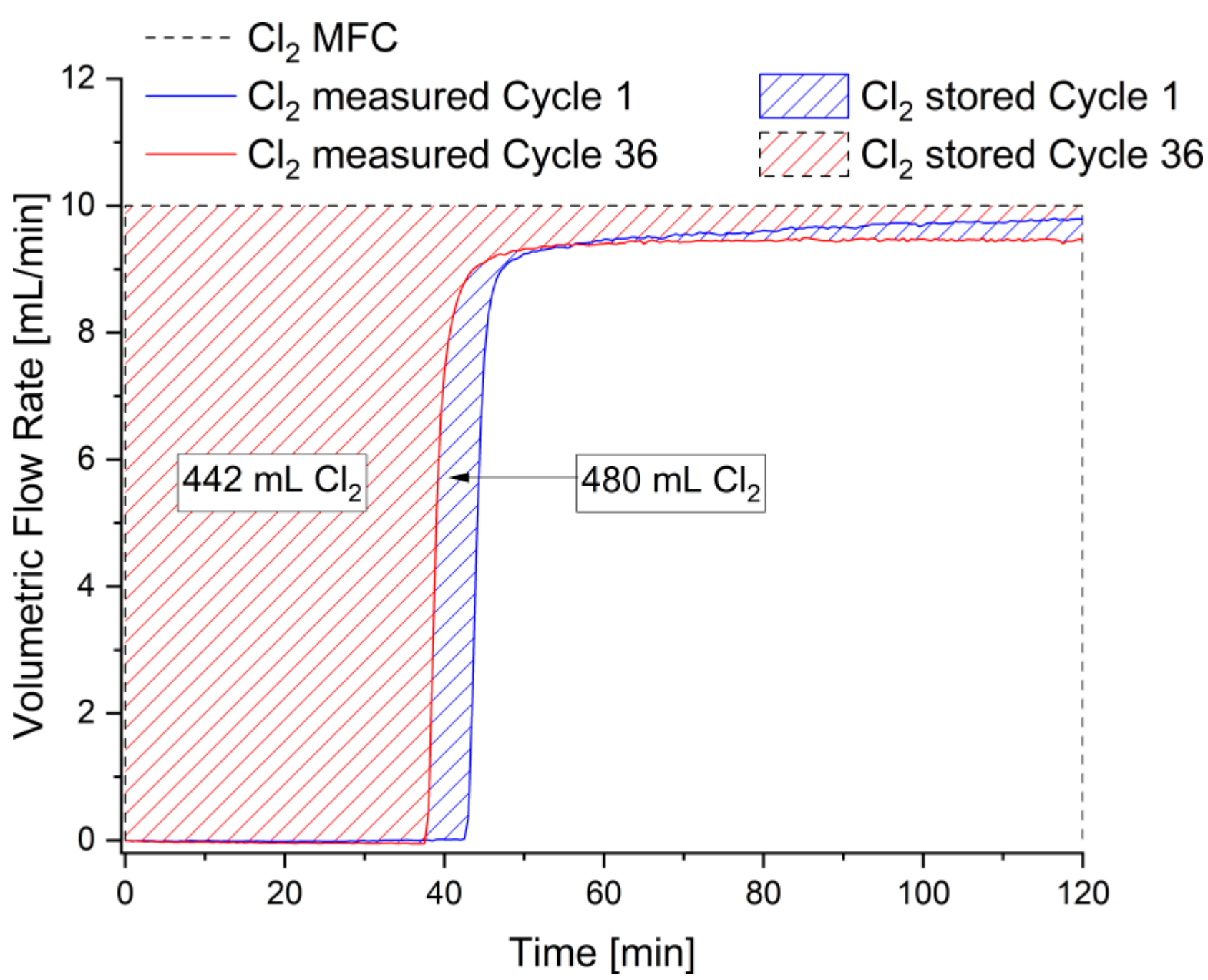

Figure S19. Change of the Cl2 storage capacity of [IEx]Cl of the first adsorption cycle (blue) and after use over a time of six months (red).

Flow Rates for the Chlorine Release by Reacting [IEx][Cl_3] with Ethylene in Flow

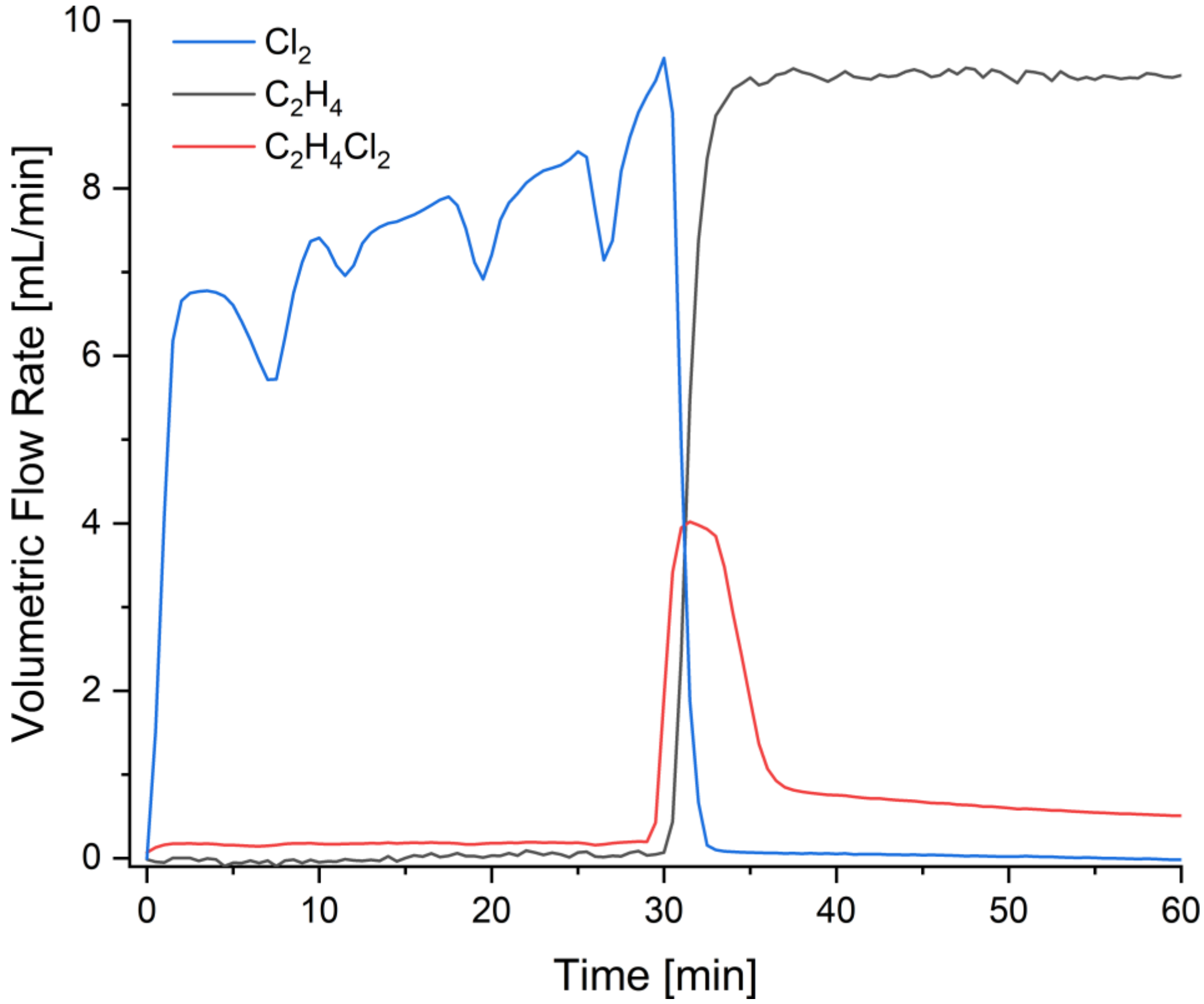

Figure S20. Determined volumetric flow rate for Cl_2, C_2H_4 and $C_2H_4Cl_2$ for an experiment were a mixture of C_2H_4 and air (7 mL/min C_2H_4, 63 mL/min Air) was pasted over the [IEx]Cl which was previously charged by passing a mixture of Cl_2 and air (10 % Cl_2 in air) over it.

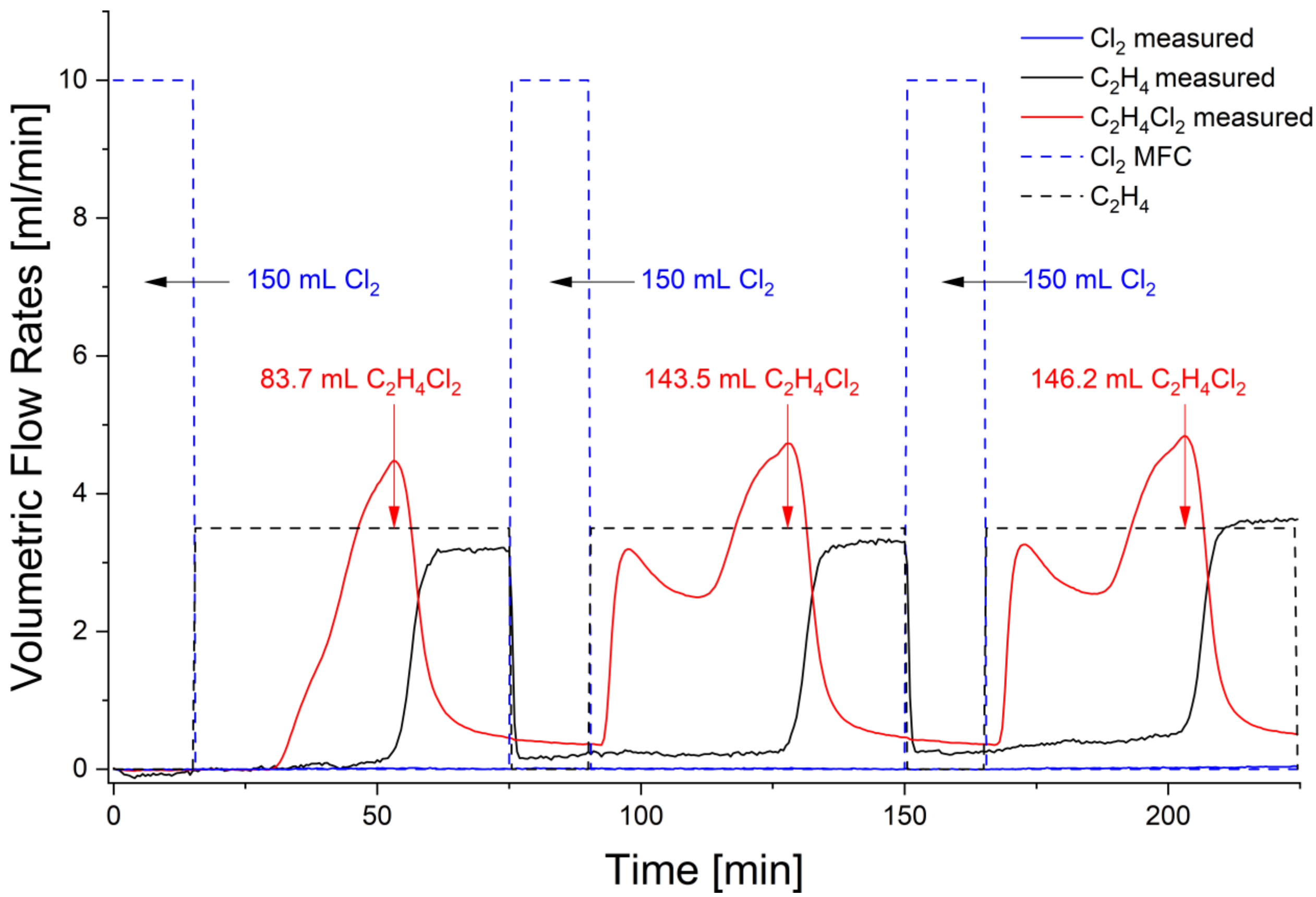

Figure S21. Three storage release cycles of [IEx]Cl were for each cycle first 150 mL of chlorine (flow rates: 10 mL/min Cl_2, 90 mL/min air) was loaded onto the polymer and then 210 ml of ethylene (flow rates 3.5 mL/min C_2H_4, 90 ml/min air) was passed over the loaded polymer. The reaction was carried at 20 °C.

Flow Rates for the Chlorine Release by Reacting [IEx][Cl_3] with CO in Flow

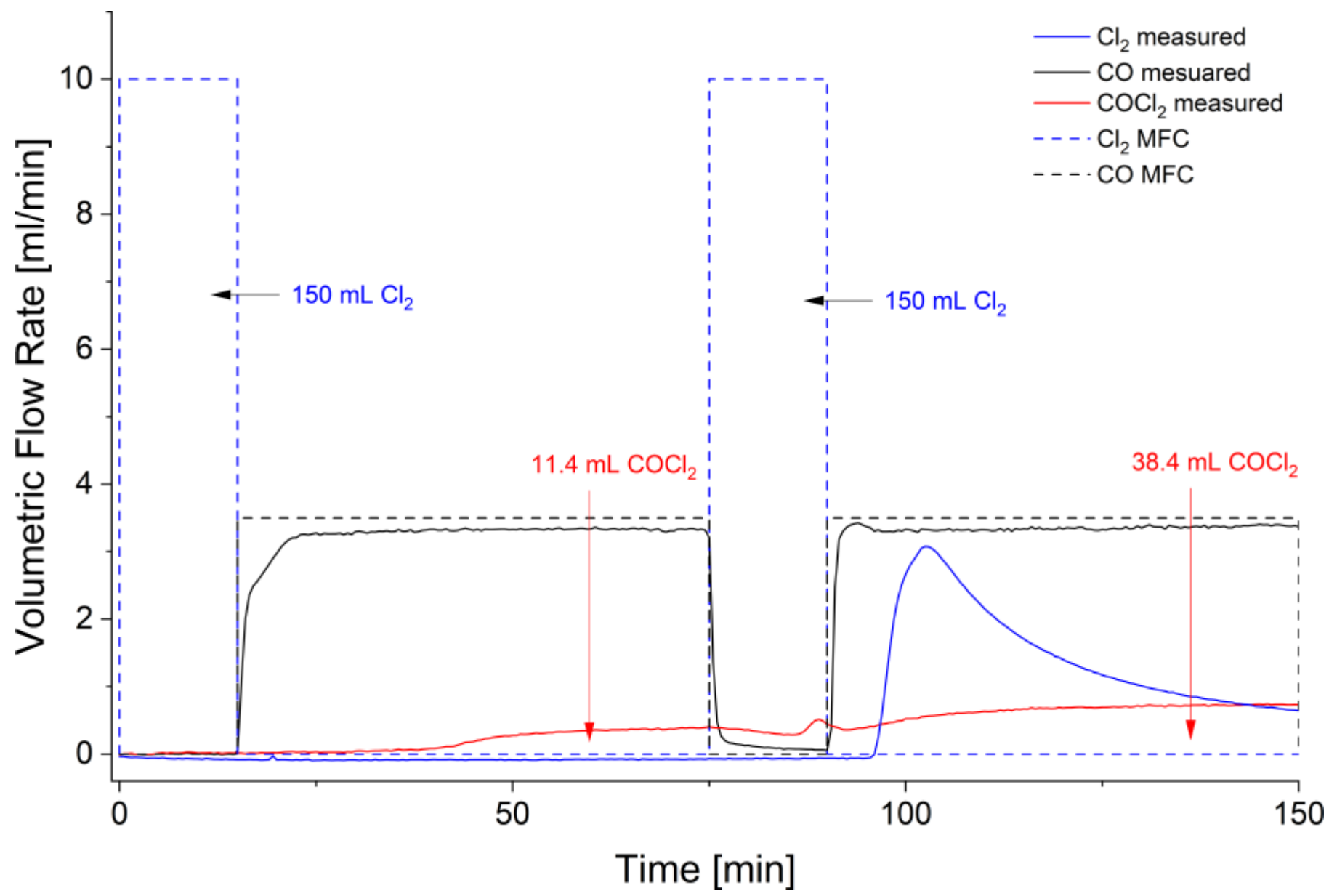

Figure S22. Two storage release cycles of [IEx]Cl were for each cycle first 150 mL of chlorine (flow rates: 10 mL/min Cl_2, 90 mL/min air) was loaded onto the polymer and then 210 ml of carbon monoxide (flow rates 3.5 mL/min CO, 90 ml/min air) was passed over the loaded polymer. The reaction was carried at 20 °C.

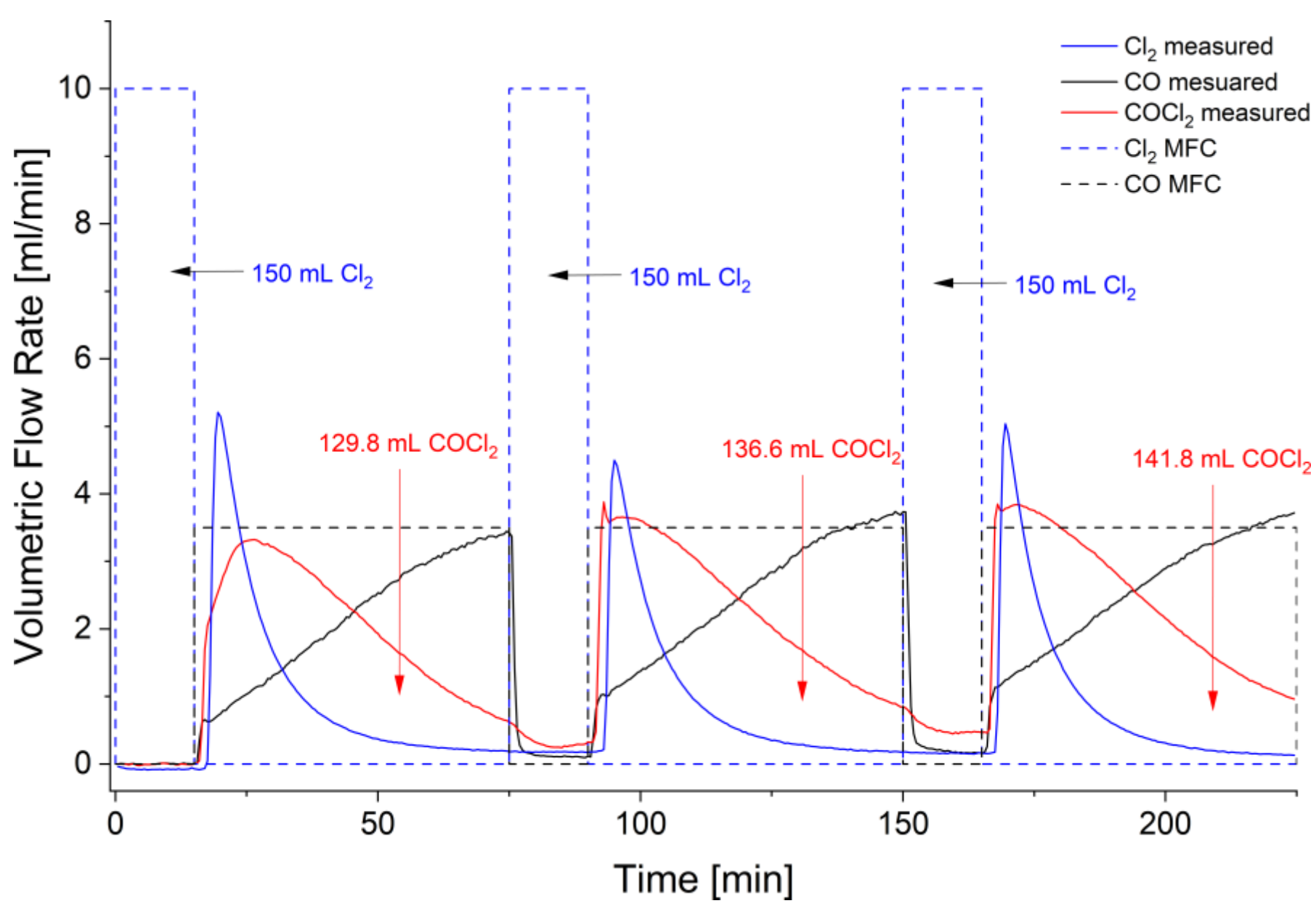

Figure S23. Three storage release cycles of [IEx]Cl were for each cycle first 150 mL of chlorine (flow rates: 10 mL/min Cl_2, 90 mL/min air) was loaded onto the polymer and then 210 ml of carbon monoxide (flow rates 3.5 mL/min CO, 90 ml/min air) was passed over the loaded polymer. The reaction was carried at 80 °C.

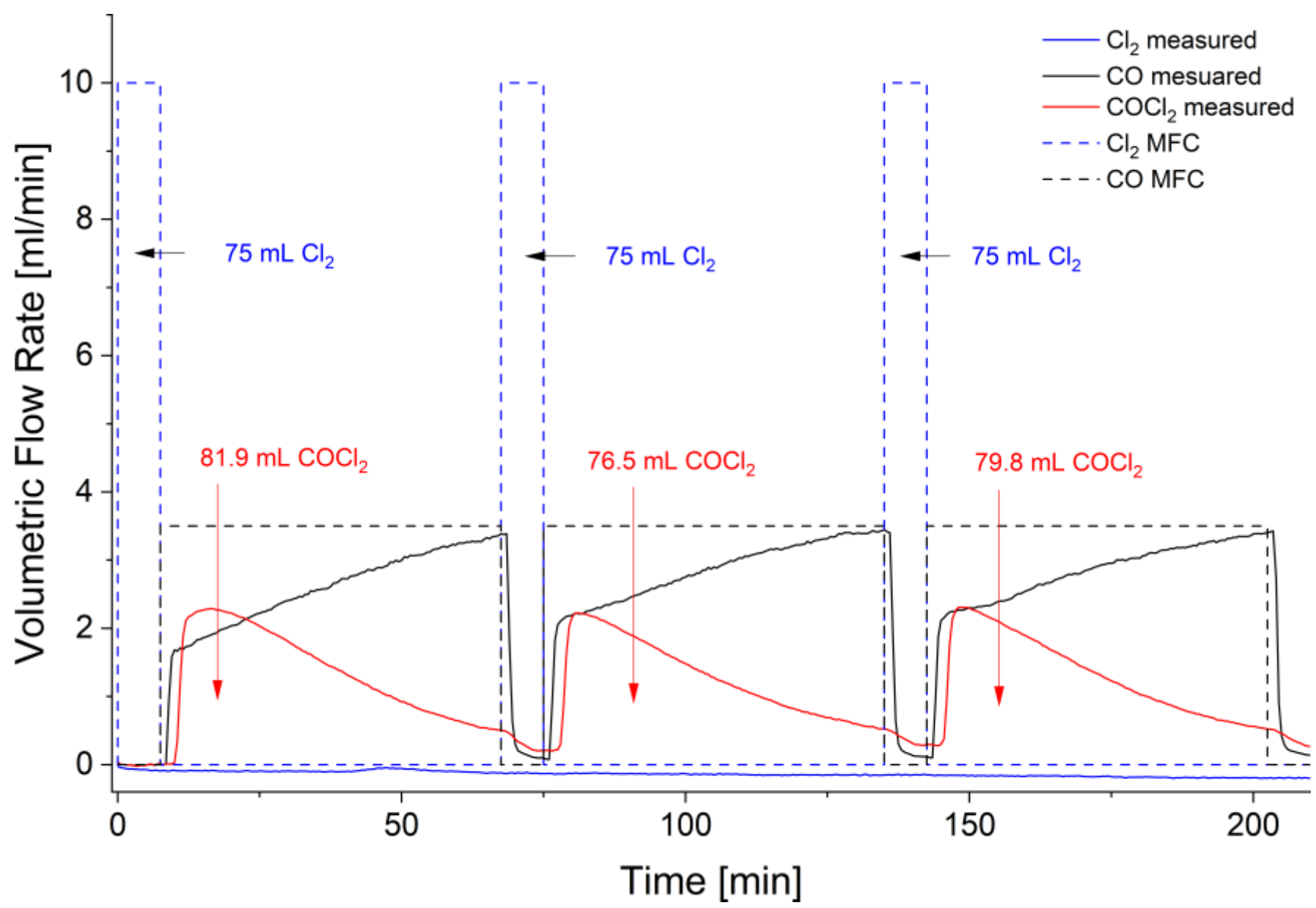

Figure S24. Three storage release cycles of [IEx]Cl were for each cycle first 75 mL of chlorine (flow rates: 10 mL/min Cl_2, 90 mL/min air) was loaded onto the polymer and then 210 ml of carbon monoxide (flow rates 3.5 mL/min CO, 90 ml/min air) was passed over the loaded polymer. The reaction was carried at 80 °C.

Heat Evolution During Chlorine Loading and Chlorine Release by Reaction with Ethylene

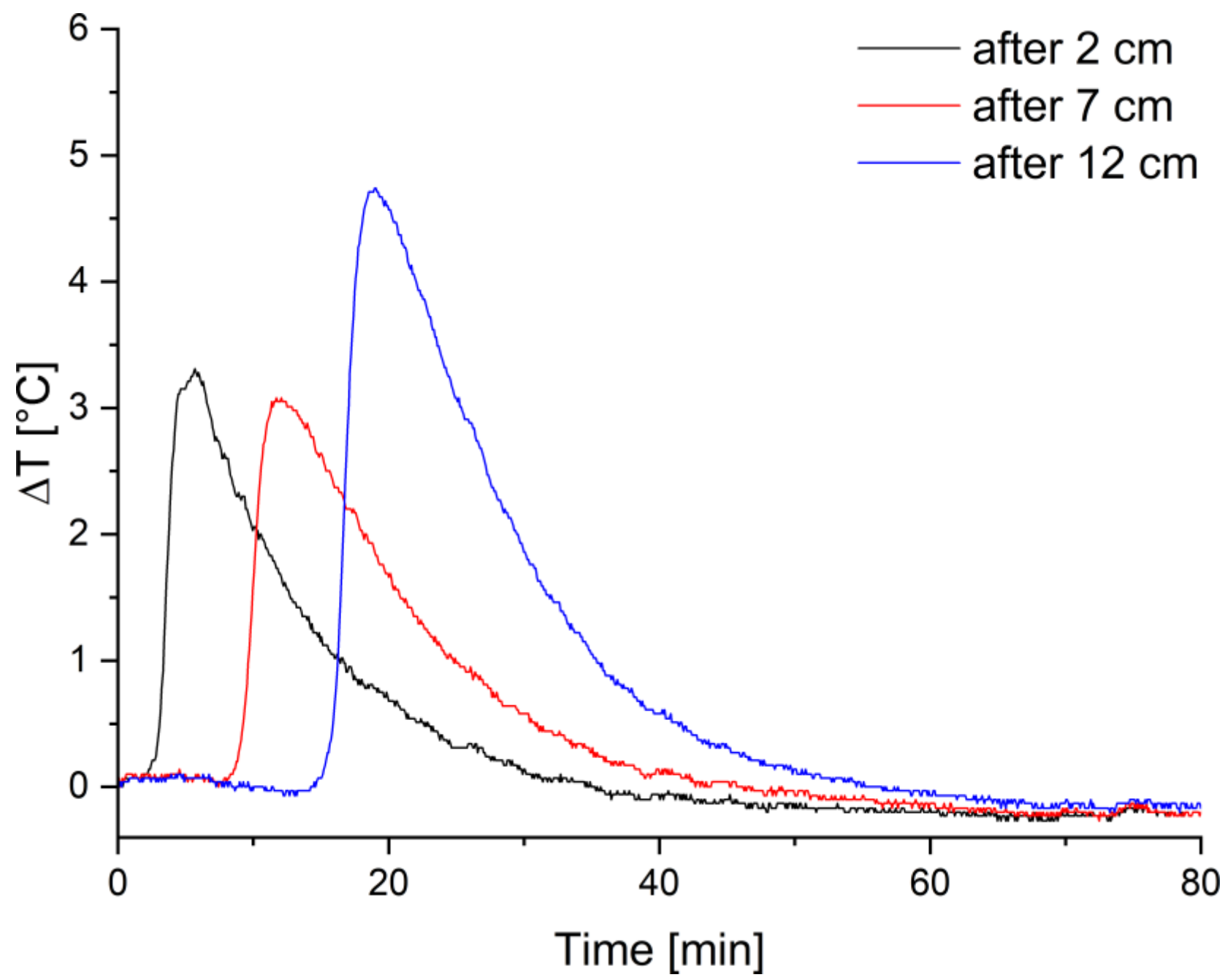

Figure S25. Temperature changes within the separation column for the loading of [IEx]Cl with chlorine (10 mL/min Cl_2 90 mL/min air). The temperature change was measured using PT100 temperature sensors place at 2, 7 and 12 cm from the top of the column. The column was not cooled for the reaction.

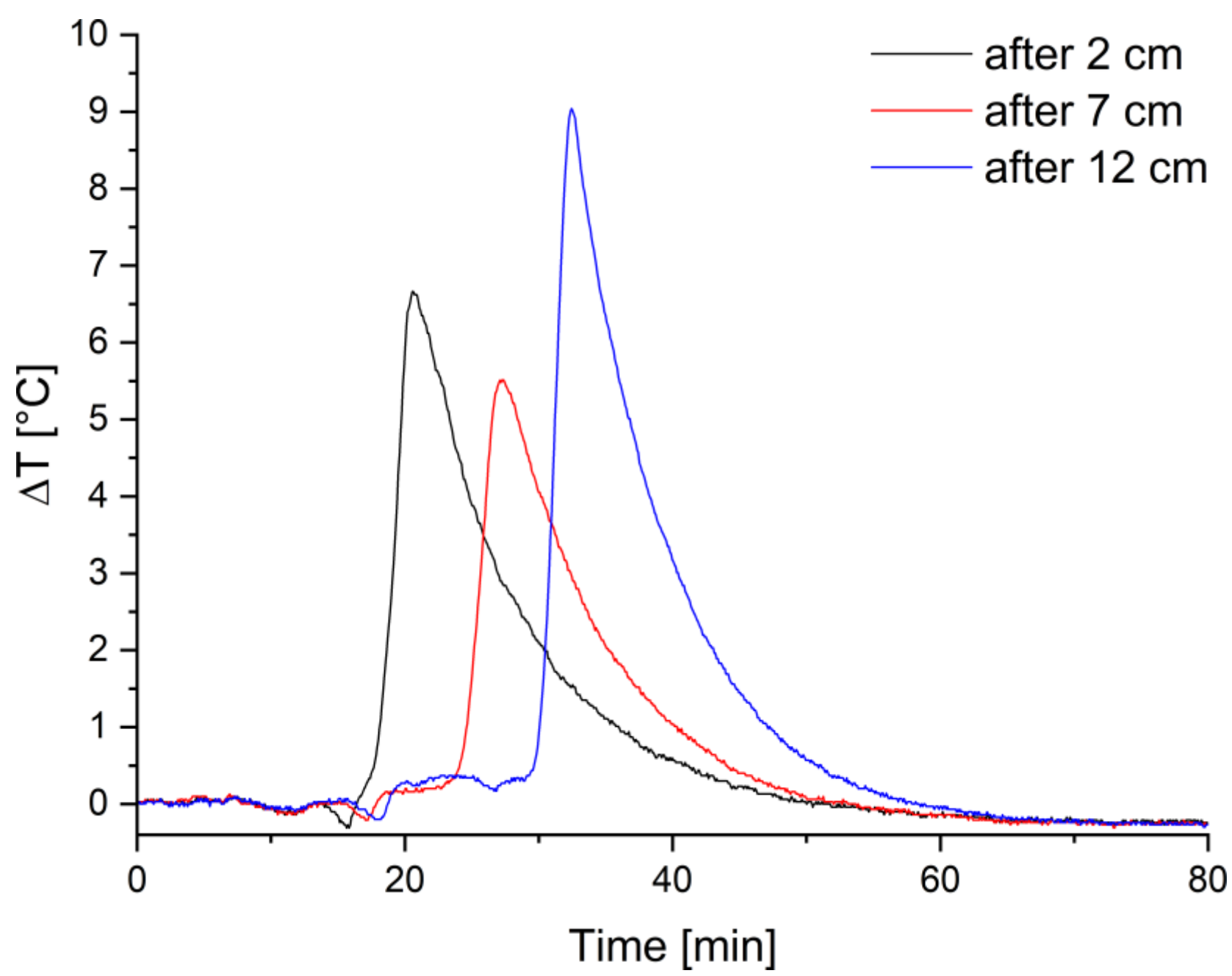

Figure S19. Temperature changes within the separation column for the reaction of [IEx][Cl_3] with ethylene (7 mL/min C_2H_4, 63 mL/min air). The temperature change was measured using PT100 temperature sensors place at 2, 7 and 12 cm from the top of the column. The column was not cooled for the reaction.

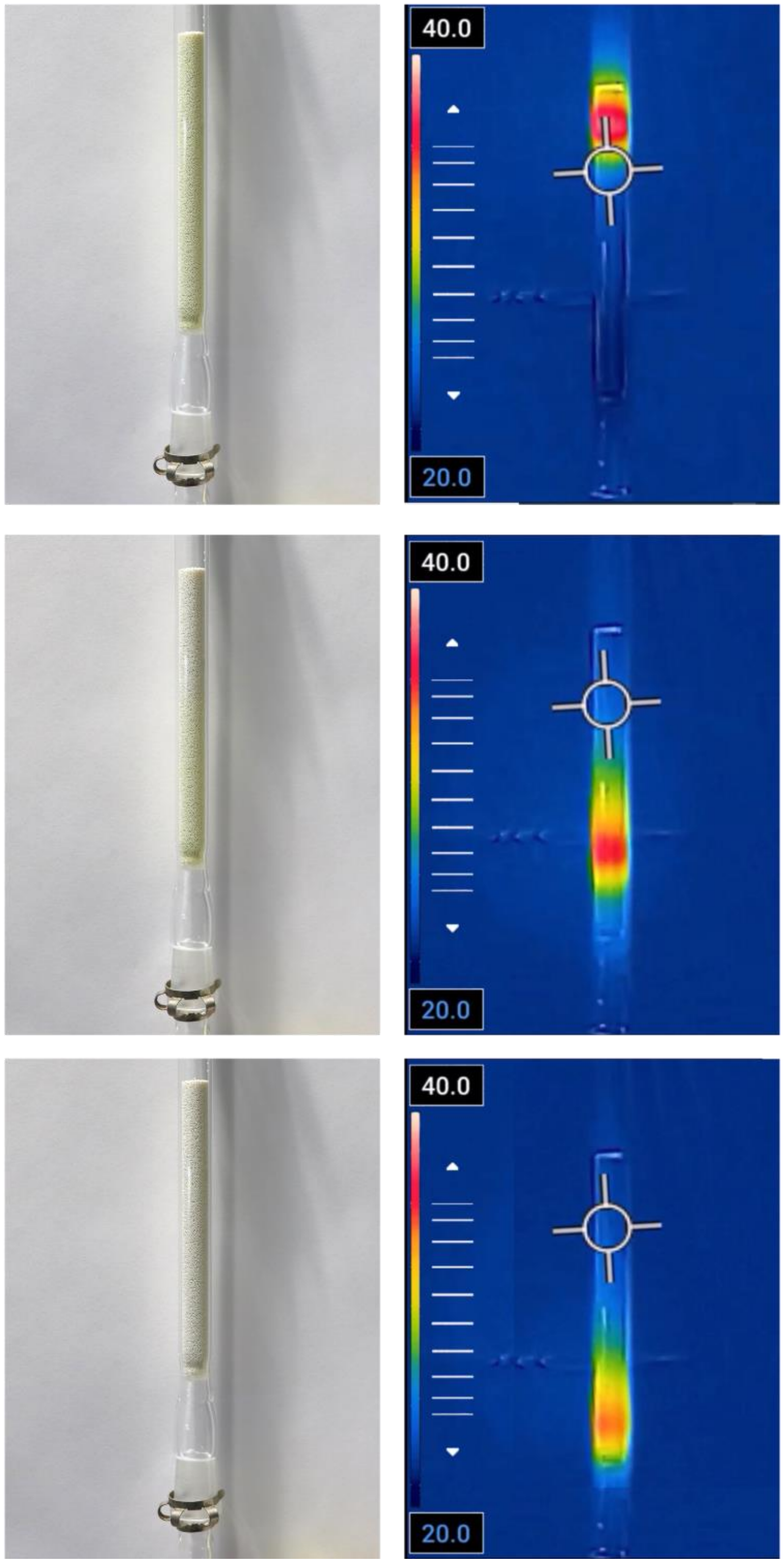

Figure S27. Chlorine release by reaction with ethylene (5 Ll/min C_2H_4, 90 mL/min air) analyzed using a thermal imaging camera revealing hotspot temperatures of 40 °C.

Calculation of Energy Consumption of European Chlorine Production

Chlorine (Cl_2) is produced by so-called chloralkali electrolysis (CAE), which consumes 2.58 MWh of electrical energy per ton of Cl_2.[2] In 2024, European energy production was at 2,732 TWh, whereas 8041 t Cl_2 have been produced resulting in:[3,4]

$$E_{Cl_2\,production} = 2.58\ \mathrm{MWh\ t^{-1}} \cdot 8{,}041{,}000\ \mathrm{t} = 20.746\ \mathrm{TWh} \tag{10}$$

$$\frac{20.746\ \mathrm{TWh}}{2{,}732\ \mathrm{TWh}} = 0.8\% \tag{11}$$

This means, that 0.8% of the European electrical energy consumption is used to produce Cl_2 by chloralkali electrolysis.

Saving Potential for Chloralkali Electrolysis

To produce 1 t of Cl_2 by CAE, approximately 2.7 MWh of electrical energy are required in Europe including the energy loss by overpotential.[5]

Currently, in Germany approx. 3.7 mio t Cl_2 are produced per year (see above), consuming

$$3{,}700{,}000\ \mathrm{t} \times 2.7\ \frac{\mathrm{MWh}}{\mathrm{t}} = 9{,}990\ \mathrm{GWh} \tag{12}$$

Given the averaged CO_2 emission of 429 g per kWh (= 429 t/GWh) in Germany,[6] the total CO_2 emission caused by the production of chlorine is

$$9{,}990\ \mathrm{GWh} \times 429\ \frac{\mathrm{t}}{\mathrm{GWh}} = 4{,}285{,}710\ \mathrm{t} \tag{13}$$

Notably, during chlorine production, a chlorine-containing tail gas stream is formed which is responsible for the loss of approximately 4% of the produced chlorine, which is quenched with NaOH.[7] In the case of a chlorine plant with an annual production volume of 300,000 t Cl_2, the chlorine production consumes

$$300{,}000\ \mathrm{t} \times 2.7\ \frac{\mathrm{MWh}}{\mathrm{t}} = 810\ \mathrm{GWh} \tag{14}$$

Considering that 4% of chlorine are lost during the production, by avoiding the chlorine loss, the electrical energy demand could be reduced to

$$\left(\frac{300{,}000\ \mathrm{t}}{1.04}\right) \times 2.7\ \frac{\mathrm{MWh}}{\mathrm{t}} = 779\ \mathrm{GWh} \tag{15}$$

This corresponds to an energy saving of 31 GWh, which translates to a CO_2 emission of

$$31\ \mathrm{GWh} \times 429\ \frac{\mathrm{t}}{\mathrm{GWh}} = 13{,}000\ \mathrm{t} \tag{16}$$

The industrial electricity price is estimated to be approximately 100 EUR per MWh. Therefore, the cost savings (based on energy savings only, value of the subsequently produced base chemicals by our technology and of NaOCl neglected) are:

$$31{,}000\ \mathrm{MWh} \times 100\ \mathrm{EUR} = 3{,}100{,}000\ \mathrm{EUR} \tag{17}$$

Considering the recycling of chlorine from other tail- and waste gas streams, the savings of energy, CO_2, and costs are more complex to calculate.

Discussion of the Bonding Situation in [IEx][Cl_3]

The Raman spectrum of the loaded polymer storage medium [IEx][Cl_3] shows an intense vibrational band at 475 cm^{-1} which is characteristic for polychloride species. The band is significantly shifted to lower wavenumbers in comparison to free chlorine which indicates a weakening of the bond within the bound chlorine molecule. This can be explained by the bonding situation in trichlorides (**Figure S28**).

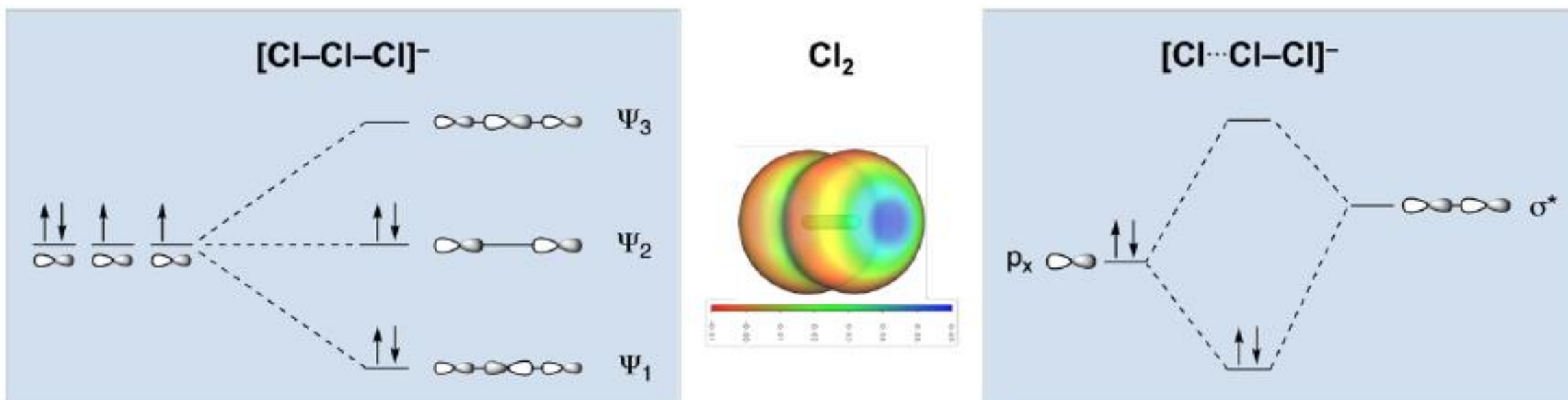

Figure S28. 1.4 Overview of different agempts to describe the bonding situation in trichlorides. Left: RUNDLE-PIMENTEL scheme to describe a three-center-four-electron bond in symmetric trichlorides ([Cl–Cl–Cl]–). Middle: Electrostatic potential of dichlorine (Cl2) in the range of –0.01 a.u. (red) to 0.06 a.u. (blue) mapped onto its electron density (isosurface value 0.0035 a.u.); calculated at the B3LYP-D3/def2-TZVPP level of theory. Right: Molecule orbital scheme of donor-acceptor interactions between a chloride anion (Cl^-) and a dichlorine molecule (Cl_2) to describe asymmetric trichlorides ([Cl…Cl–Cl]–).[8] Reproduced from Ref. 8 with permission of Wiley-VCH Verlag GmbH & Co. KGaA.

For the symmetric $[Cl_3]^-$ anion the bonding situation can best be described as a three-center-four-electron (3c-4e) bond with one bonding (Ψ_1), one non-bonding (Ψ_2) and one antibonding (Ψ_3) molecular orbital (**Figure S27**, left). This symmetric bonding results in a bond order of 0.5 and therefore a strong shift in the Raman spectrum compared to free chlorine. On the other hand, asymmetric $[Cl_3]^-$ anions can best be described by a donor-acceptor interaction between the Lewis-basic chloride anion and a Lewis-acidic Cl_2 molecule (**Figure S27**, right). The bonding is achieved by a charge transfer from the p-orbital of the chloride anion into the σ*-orbital of Cl_2 molecule also resulting in weakening of the Cl_2 bond as electron density is donated into an orbital with antibonding character. However, this weakening is less pronounced compared to symmetric trichlorides which also leads to a less pronounced shift of the Raman band. Asymmetric trichlorides are usually observed when the chloride ion is stabilized by additional interactions e.g. hydrogen-bonding as expected for the interaction between the polymer backbone of the ion exchange resin and the chloride ions.

Movie S1: Loading Chlorine

In this Movie S1 the loading process of [IEx]Cl with gaseous Cl_2 is shown. The Cl_2 propagation can be observed by the color change of the polymer to pale yellow and by the thermographic image showing the heat generation.

Movie S2: Release Ethylene

In this Movie S2 the reactive release of Cl_2 from [IEx]Cl by its conversion to 1,2-dichloroethane using gaseous ethylene is shown. The reaction process can be observed by the color change of the polymer to beige and by the thermographic image showing the heat generation.

References and Notes

[1] P. Voßnacker, A. Wüst, T. Keilhack, C. Müller, S. Steinhauer, H. Becker, S. Yogendra, Y. Schiesser, R. Weber, M. Reimann, R. Müller, M. Kaupp, S. Riedel, *Sci. Adv.* **2021**, *7*, eabj5186.

[2] M. Kleoff, F. Klaucke, P. Voßnacker, R. Weber, K. Neuhoff, S. Riedel, *Green Chem.* **2025**, *27*, 9874-9881.

[3] Eurelectric, *Power Barometer 2025*, https://powerbarometer.eurelectric.org/#:~:text=And%20yet%2C%20Europe%20remains%20firmly,modest%20gain%20of%207%20TWh (accessed: 08.03.2026).

[4] EuroChlor, *Chlor-alkali industry review 2024-2025*, https://www.eurochlor.org/wp-content/uploads/2025/09/Chlor-Alkali-Industry-Review-2024_2025_FINAL-CORRECTED-2025-08-26.pdf (accessed: 08.03.2026).

[5] https://www.statista.com/statistics/550212/electricity-production-uk/ (accessed: 15.11.2024).

[6] https://www.eurochlor.org/wp-content/uploads/2018/06/12-Electrolysis-production-costs-November-2023.pdf (accessed: 15.11.2024).

[7] https://www.umweltbundesamt.de/themen/klima-energie/energieversorgung/strom-waermeversorgung-in-zahlen#Strommix (accessed: 15.11.2024).

[8] K. Sonnenberg, L. Mann, F. A. Redeker, B. Schmidt, S. Riedel, *Angew. Chem. Int. Ed.* **2020**, *59*, 5464-5493.
